# Conformational change of adenine nucleotide translocase‐1 mediates cisplatin resistance induced by EBV‐LMP1

**DOI:** 10.15252/emmm.202114072

**Published:** 2021-11-09

**Authors:** Lin Zhao, Xiangying Deng, Yueshuo Li, Jianmin Hu, Longlong Xie, Feng Shi, Min Tang, Ann M Bode, Xin Zhang, Weihua Liao, Ya Cao

**Affiliations:** ^1^ Key Laboratory of Carcinogenesis and Cancer Invasion Chinese Ministry of Education, Department of Radiology Xiangya Hospital Central South University Changsha China; ^2^ Cancer Research Institute and School of Basic Medical Science Xiangya School of Medicine Central South University Changsha China; ^3^ Key Laboratory of Carcinogenesis Chinese Ministry of Health Changsha China; ^4^ Molecular Imaging Research Center of Central South University Changsha China; ^5^ Research Center for Technologies of Nucleic Acid‐Based Diagnostics and Therapeutics Hunan Province Changsha China; ^6^ National Joint Engineering Research Center for Genetic Diagnostics of Infectious Diseases and Cancer Changsha China; ^7^ The Hormel Institute University of Minnesota Austin MN USA; ^8^ Department of Otolaryngology Head and Neck Surgery Xiangya Hospital Central South University Changsha China; ^9^ Department of Radiology Xiangya Hospital Central South University Changsha China

**Keywords:** ANT1, chemosensitivity, conformational change, LMP1, mitochondrial membrane potential, Cancer, Organelles

## Abstract

Adenine nucleotide translocase‐1 (ANT1) is an ADP/ATP transporter protein located in the inner mitochondrial membrane. ANT1 is involved not only in the processes of ADP/ATP exchange but also in the composition of the mitochondrial membrane permeability transition pore (mPTP); and the function of ANT1 is closely related to its own conformational changes. Notably, various viral proteins can interact directly with ANT1 to influence mitochondrial membrane potential by regulating the opening of mPTP, thereby affecting tumor cell fate. The Epstein–Barr virus (EBV) encodes the key tumorigenic protein, latent membrane protein 1 (LMP1), which plays a pivotal role in promoting therapeutic resistance in related tumors. In our study, we identified a novel mechanism for EBV‐LMP1‐induced alteration of ANT1 conformation in cisplatin resistance in nasopharyngeal carcinoma. Here, we found that EBV‐LMP1 localizes to the inner mitochondrial membrane and inhibits the opening of mPTP by binding to ANT1, thereby favoring tumor cell survival and drug resistance. The ANT1 conformational inhibitor carboxyatractyloside (CATR) in combination with cisplatin improved the chemosensitivity of EBV‐LMP1‐positive cells. This finding confirms that ANT1 is a novel therapeutic target for overcoming cisplatin resistance in the future.

## Graphical abstract

EBV‐encoded tumorigenic protein LMP1 regulates conformational changes of mitochondrial protein adenine nucleotide translocase‐1 (ANT1), and thereby chemoresistance of tumor cells.

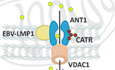

**The paper explained**

**Problem**

The Epstein–Barr virus (EBV) encodes the key oncogenic protein LMP1, which plays an important role in promoting resistance to therapy. In this study, we explored novel targets and potential mechanisms by which EBV‐LMP1 regulates resistance to cisplatin in nasopharyngeal carcinoma (NPC).

**Results**

We found that EBV‐LMP1 can localize to the mitochondria to bind directly to adenine nucleotide translocase‐1 (ANT1), fixing the ANT1 conformation in the m‐state, thereby increasing the mitochondrial membrane potential and promoting the viability of NPC cells. Carboxyatractyloside (CATR), a conformational inhibitor of ANT1, which contributes to mPTP opening and cell death, enhanced the sensitivity of tumor cells to cisplatin.

**Impact**

Our study links for the first time ANT1 conformational changes to cisplatin chemosensitivity, highlighting the importance of protein conformational changes in tumor chemotherapy.

## Introduction

Adenine nucleotide translocase‐1 (ANT1) is a mitochondrial inner membrane protein located in the mitochondria and is responsible for mitochondrial ADP/ATP transport (Parodi‐Rullaán *et al*, 2019; Ruprecht & Kunji, 2020). A recent study confirms that ANT1 is a key protein driving mitochondrial autophagy (Hoshino *et al*, 2019), while ANT is involved in proton transport in the presence of fatty acids (Bertholet *et al*, 2019). Adenine nucleotide translocase‐1 is also a major component of the mitochondrial membrane permeability transition pore (mPTP) (Bround *et al*, 2020). Protein complexes of ANT with VDAC and cyclophilin D play a crucial role in maintaining the mitochondrial membrane potential (Δψm) and permeability (Bertholet *et al*, 2019; Ruprecht *et al*, 2019). Despite extensive work, the molecular composition of mPTP is currently not fully understood and remains an area of debate, with ANT and F0F1‐ATP synthase being the main contenders for its components (Brustovetsky, 2020). ANT has two different conformations: When ANT1 is facing the mitochondrial matrix, it transports ATP to release ADP, which is in the m‐state, and when ANT1 is facing the cytoplasmic side, it transports ADP to release ATP, which is in the c‐state; ANT achieves mitochondrial energy conversion through a cyclic state transition, the inhibitors CATR and bongkrekic acid (BKA) fix ANT1 in the c‐state and m‐state, respectively (Bertholet *et al*, 2019; Ruprecht *et al*, 2019; Ruprecht & Kunji, 2020). The c‐state of ANT has been found to be an important condition for mPTP opening, suggesting that transitions between conformations of ANT are not only critical for mitochondrial synthesis of ATP but also key mechanisms for maintaining the fate of cancer cells (Novgorodov *et al*, 1992; Halestrap & Brenner, 2003; Ruprecht *et al*, 2019; Zhao *et al*, 2021).

Several viral proteins have been shown to interact directly with ANT1 to regulate mPTP function. For example, the cytomegalovirus protein (vMIA) can inhibit the opening of mPTP by interacting with ANTs, and the human immunodeficiency virus‐1 (HIV‐1) encoding viral protein R (Vpr) can bind to ANTs to induce mPTP‐driven apoptosis (Jacotot *et al*, 2001; Vieira *et al*, 2001; Sabbah *et al*, 2006; Tanaka *et al*, 2008; Green *et al*, 2011; Wang *et al*, 2017). The Epstein–Barr virus (EBV) is the first DNA virus determined to be tumorigenic and persistently infectious in humans, resulting in approximately 95% of the world's asymptomatic infections. It is associated with multiple cancers, including nasopharyngeal carcinoma (NPC) (Chou & Talalay, 1983; Lieberman, 2014; Young *et al*, 2016; Chen *et al*, 2019). EBV encodes the key oncogenic protein, latent membrane protein 1 (LMP1), which plays a central role in promoting therapeutic resistance in related tumors (Shair *et al*, 2018; Shi *et al*, 2019; Xie *et al*, 2020). Cisplatin is the most important and classic first‐line synchronous chemotherapeutic agent for the treatment of nasopharyngeal carcinoma; and EBV‐LMP1 regulates cellular cisplatin resistance through multiple signaling pathways (Tang *et al*, 2018). Inhibition of AKT after LMP1 knockdown enhances tumor cell sensitivity to cisplatin; and LMP1 regulates mitochondrial dynamic protein‐related protein 1 (Drp1) to promote NPC cell survival and cisplatin resistance (Mei *et al*, 2007; Xie *et al*, 2020). In addition, the pivotal mitochondrial protein ANT1 showed increased sensitivity to cisplatin after knockdown in non‐small cell lung cancer (Tajeddine *et al*, 2008). These findings suggest that ANT1 may be an important cause of LMP1‐triggered tumor drug resistance, which is not yet fully understood.

Our findings provide novel insights into viral protein regulation of ANT1 conformational changes affecting mitochondrial function and tumor drug resistance. Here, we show for the first time that EBV‐LMP1 localizes to the inner mitochondrial membrane to directly interact with ANT1, resulting in a block of the mPTP opening and elevated mitochondrial membrane potential, thereby increasing tumor cell viability. The specific mechanism involves an inhibition of the conformational change of ANT1 by EBV‐LMP1, which decreases the transport activity of ANT1 and attenuates the formation of the ANT1‐VDAC1 complex. In addition, the ANT1 conformation inhibitor, CATR, reversed this process, resulting in a significant increase in the sensitivity of NPC cells to cisplatin. Thus, a molecular link between LMP1 and cisplatin resistance was established in which the inhibition of ANT1 conformation by EBV‐LMP1 was a determinant of reduced cisplatin sensitivity in tumor cells.

## Results

### EBV‐LMP1 inhibits mPTP opening to increase the mitochondrial membrane potential (Δψm)

**Figure 1:**
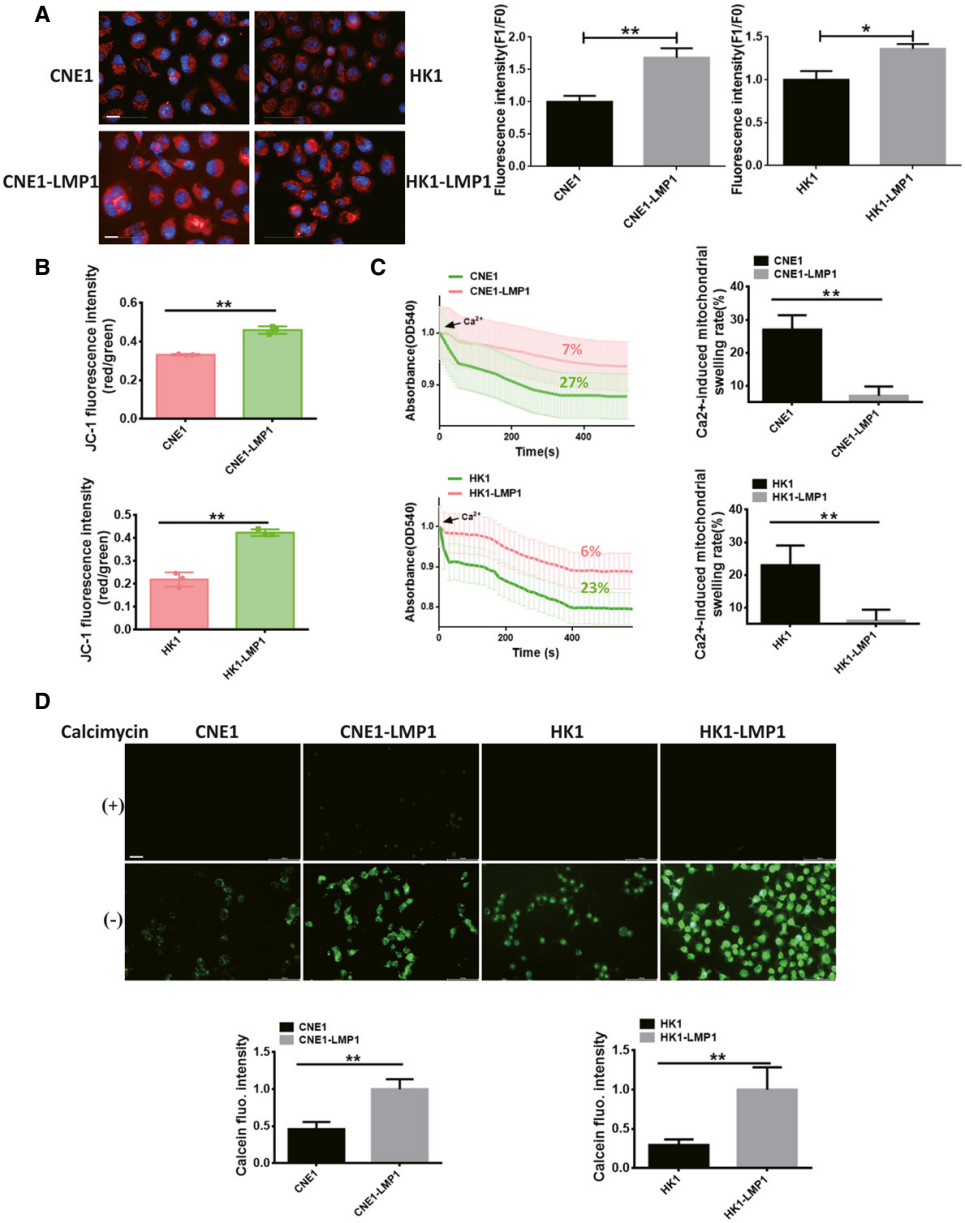
EBV‐LMP1 inhibits mPTP opening and increases Δψm Confocal observation of mitochondrial potential after TMRM staining of LMP1‐negative or LMP1‐positive NPC cells. Red fluorescence, which indicates normal mitochondrial potential, was converted into green fluorescence after a reduction in mitochondrial potential. Images were analyzed using ImageJ software (scale bar, 10 μm). Data are presented as means ± SEM (paired *t*‐test, *n* = 6, biological replicates per group, *P < 0.05, **P < 0.01).The mitochondrial potential was detected by JC‐1 staining. Data are presented as means ± SEM (paired *t*‐test, *n* = 3, biological replicates per group, **P < 0.01).Extent of Ca^2+^‐mediated mitochondrial swelling in NPC cells. Data are presented as means ± SEM (paired *t*‐test, *n* = 6, biological replicates per group, **P < 0.01).The extent of mPTP opening in nasopharyngeal carcinoma cells (scale bar, 20 μm). Calcimycin (calcium ionophore) served as a positive control. Images were analyzed using ImageJ software. Data are presented as means ± SEM (paired *t*‐test, *n* = 6, biological replicates per group, **P < 0.01).

To understand the differences in membrane potential in LMP1‐negative or LMP1‐positive NPC cells, two different Δψm sensitive probes, TMRM (tetramethyl rhodamine methyl ester) and JC‐1, were used. Two sets of NPC cells, CNE1/CNE1‐LMP1 and HK1/HK1‐LMP1, were stained with TMRM and then observed by laser confocal microscopy to show that the fluorescence intensity of CNE1‐LMP1 and HK1‐LMP1 cells was significantly increased compared with CNE1 and HK1 parental cells (Fig 1A). The JC‐1 assessment further confirmed that the mitochondrial membrane potential of LMP1‐positive NPC cells increased substantially; and the difference was statistically significant (Fig 1B).

Under normal physiological conditions, the mPTP allows free passage of solutes ≤ 1.5 KD; but in some pathological conditions the mPTP opens, mitochondria undergo swelling, and the Δψm decreases, eventually leading to cell death (Bonora *et al*, 2015; Fricker *et al*, 2018). Therefore, an analysis of the degree of mitochondrial swelling could be important in examining changes in the mitochondrial membrane potential. Next, we investigated the effect of EBV‐LMP1 on Ca^2+^‐mediated mitochondrial swelling by extracting mitochondria from the two sets of NPC cells separately. The mitochondrial swelling rates of CNE‐LMP1 (7%) and HK1‐LMP1 (6%) cells were significantly lower than those of CNE1 (27%) and HK1 (23%) cells, suggesting that EBV‐LMP1 decreases Ca^2+^‐mediated mitochondrial swelling and increases the mitochondrial membrane potential (Fig 1C).

We further used the calcein‐AM probe to detect the opening of mPTP in the two sets of NPC cells. The reduced fluorescence compared to the initial fluorescence amount represents the degree of mPTP openness (Fig 1D). In these two sets of NPC cells, the openness of mPTP in LMP1‐positive NPC cells was significantly reduced compared to the control group. The above results suggest that EBV‐LMP1 increases the mitochondrial potential by inhibiting mPTP opening.

**Figure EV1:**
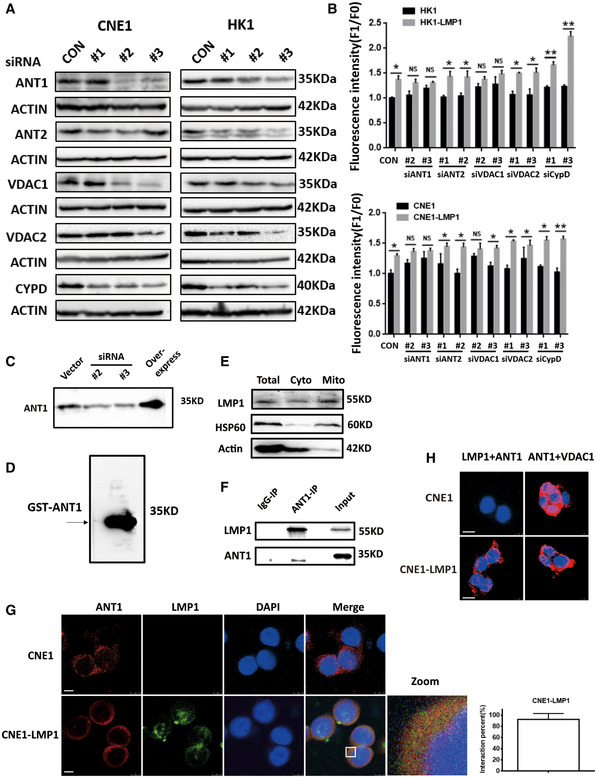
EBV‐LMP1 interacting with ANT1 localizes to mitochondria AKnockdown effect of mPTP complex components.BEffect of knockdown of each mPTP component on the membrane potential of EBV‐LMP1‐negative or EBV‐LMP1‐positive nasopharyngeal carcinoma cells. Data are presented as means ± SEM (paired *t*‐test, *n* = 5, biological replicates per group, *P < 0.05, **P < 0.01).C, DThe specificity of ANT1 antibody was verified in this experiment using: knockdown or overexpression of ANT1 in HK1 cells, respectively (C); expression of HK1‐derived ANT1 in prokaryotes and validation after purification (D).ELMP1 is present in the cytoplasm and mitochondria. EBV‐LMP1‐positive nasopharyngeal carcinoma cells were isolated from cytoplasm and mitochondria and western blot assayed for LMP1 expression.FCo‐IP detection of the interaction of EBV‐LMP1 and ANT1 in CNE1‐LMP1 cells.GLaser confocal analysis of CNE1‐LMP1 cells revealed the presence of co‐localization of LMP1 with ANT1 (scale bar, 5 μm). The quantified graph on the lower right shows the percentage of yellow fluorescence (merge) to red fluorescence (ANT1) in CNE1‐LMP1 cells. Data are presented as means ± SEM (*n* = 6, biological replicates per group).HPLA detects the presence of direct binding of LMP1 to ANT1, but not VDAC1, in CNE1‐LMP1 cells (scale bar, 10 μm).

The main components of mPTP consist of VDAC1/2 at the outer membrane, ANT1/2/3 at the inner membrane, and CypD at the matrix (Wang *et al*, 2014). We first examined the efficiency of VDAC1/2, ANT1/2, and CypD knockdown (Fig EV1A). Subsequently, we compared the differences in cell membrane potential changes: after VDAC2/ANT2/3 and CypD knockdown, the membrane potential of CNE1‐LMP1 and HK1‐LMP1 cells was significantly higher than that of CNE1 and HK1 (Fig EV1B). In contrast, no statistically significant difference was observed between VDAC1 and ANT1 knockdown (Fig EV1B). These results suggest that VDAC1 and ANT1 affect the regulation of mitochondrial membrane potential by LMP1, which in turn affects mPTP opening. We confirmed the specificity of ANT1 antibody by both prokaryotic and eukaryotic methods: First, we overexpressed and knocked down ANT1 in HK1 cells and then detected ANT1 expression by WB (Fig EV1C); second, we expressed HK1 cell‐derived ANT1 in prokaryotic cells, purified it, and detected it by ANT1 antibody (Fig EV1D).

### EBV‐LMP1 localizes mitochondrial endosomes to interact with ANT1

**Figure 2:**
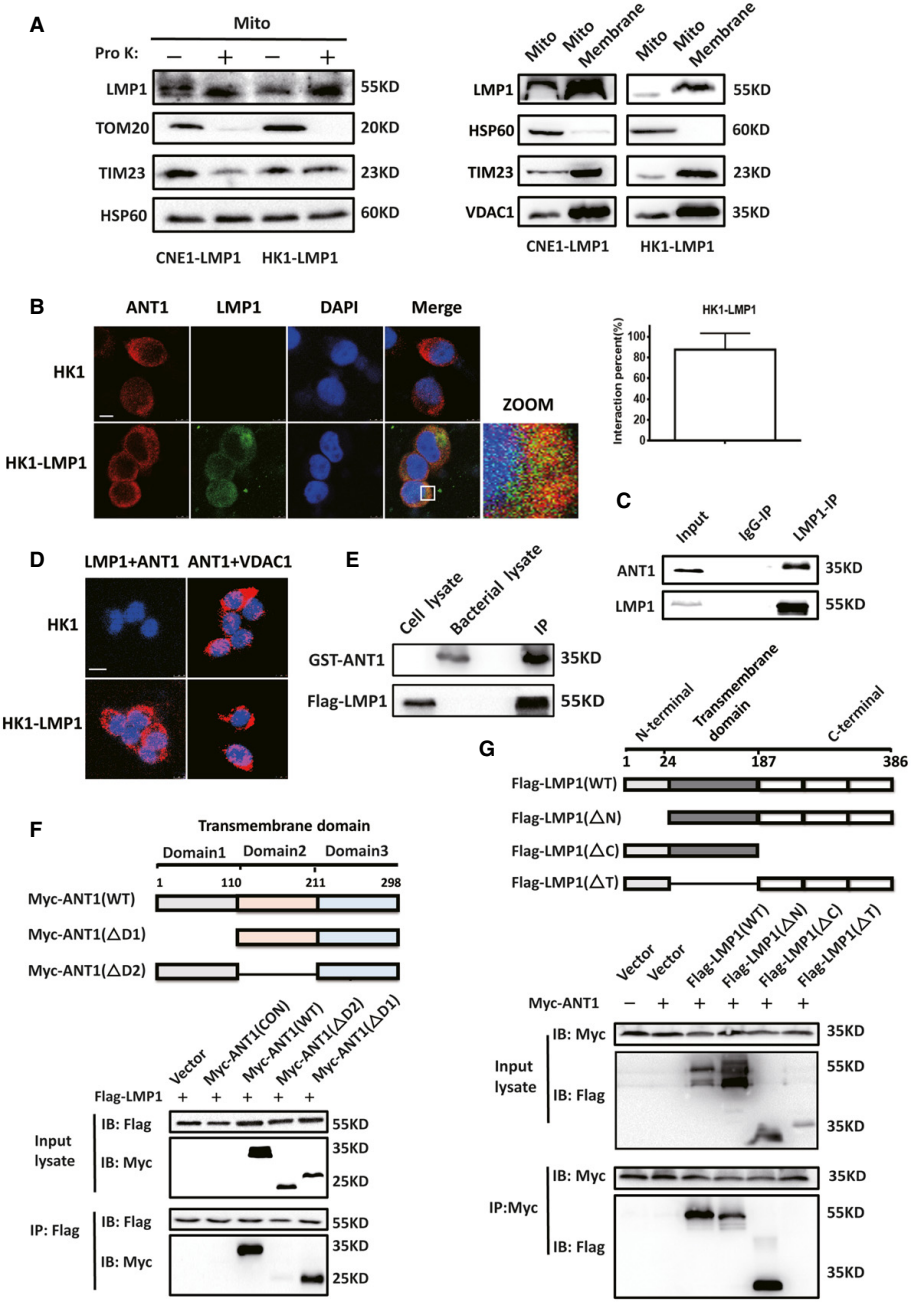
LMP1 localizes to the mitochondrial inner membrane and interacts with ANT1 in NPC cells LMP1 is localized to the inner mitochondrial membrane. Isolated mitochondria were incubated with proteinase K (40 μM) for 30 min before LMP1 expression was detected (left); the mitochondria were further subjected to submitochondrial fractionation. VDAC1, TIM23, and HSP60 were used to represent MOM, MIM, and mitochondrial matrix protein, respectively (right).The interaction of LMP1 and endogenous ANT1 was analyzed by an immunofluorescence confocal assay. HK1‐LMP1 cells were immunostained with anti‐LMP1 (green) and anti‐ANT1 (red) antibodies and then subjected to confocal microscopy, and use ImageJ to calculate the co‐location level of both. The nuclei were stained with DAPI (scale bar, 5 μm). The quantified graph on the top right shows the percentage of yellow fluorescence (merge) to red fluorescence (ANT1) in HK1‐LMP1 cells. Data are presented as means ± SEM (*n* = 6, biological replicates per group).IP/IB analysis was used to detect the interaction of LMP1 with endogenous ANT1 in CNE1‐LMP1‐expressing cells.Detection of the LMP1‐ANT1 interaction using a proximity ligation assay. Red fluorescence corresponds to the PLA‐positive signal and blue fluorescence corresponds to nuclei (DAPI staining; scale bar, 10 μm).LMP1 interaction with ANT1 *in vitro*. GST‐ANT1 expressed in bacteria was incubated with a Flag‐LMP1 protein and the proteins were subjected to Western blot analysis.ANT1 interaction with LMP1 is dependent on its domain 2 in 293T cells. ANT1 and LMP1 domain architecture and schematic representation of its respective deletion constructs used in this study in 293T cells. IP/IB was used to detect the interaction between Flag‐tagged LMP1 and Myc‐tagged ANT1 mutants. IP of cell lysates using a Flag or Myc antibody.LMP1 binds directly to ANT1 dependent on its transmembrane domain in 293T cells. IP/IB was used to detect the interaction between Flag‐tagged LMP1 mutants and Myc‐tagged ANT1. IP of cell lysates using a Flag or Myc antibody.

The previous section showed that the difference in mPTP opening leads to different membrane potentials in LMP1‐positive cells. The question is why would this difference be caused in LMP1‐positive tumor cells and does LMP1 cause this change by directly regulating ANT1 or VDAC1. To answer these questions, we first needed to determine whether LMP1 is present in mitochondria. We isolated cytosolic and mitochondrial proteins and Western blot results confirmed that LMP1 is present in both cytoplasm and mitochondria (Fig EV1E). Using proteinase K to remove the outer mitochondrial membrane (OMM) protein prevented LMP1 from remaining on the OMM (Fig 2A). Further separation of the mitochondrial membrane and matrix revealed that LMP1 was mainly present on the inner mitochondrial membrane (Fig 2A).

We further determined whether LMP1 localizes to the inner mitochondrial membrane and interacts directly with ANT1. We observed the presence of co‐localization between LMP1 and ANT1 in HK1‐LMP1 and CNE1‐LMP1cells (Figs 2B and EV1G). EBV‐LMP1 binding to ANT1 was further confirmed by immunoprecipitation (Figs 2C and EV1F). In addition, the existence of direct binding of EBV‐LMP1 to ANT1 was confirmed by an *in vitro* pull‐down assay and prokaryotic expression of GST‐ANT1 (Fig 2E). We next performed an *in situ* proximity ligation assay (PLA) in LMP1‐positive NPC cells transiently co‐express Flag‐LMP1 and Myc‐ANT1 or Myc‐VDAC1. Similar to the above results, there was a positive fluorescent signal observed in cells co‐expressing Flag‐LMP1 and Myc‐ANT1, while such a signal was not observed in cells co‐expressing Flag‐LMP1 and Myc‐VDAC1 (Figs 2D and EV1H), indicating a specific interaction between LMP1 and ANT1 in this cell compartment.

The EBV‐LMP1 protein consists of 386 amino acid residues and includes the N‐terminal, six transmembrane domains, and the C‐terminal activation domain, localized to the plasma membrane and endoplasmic reticulum (Lee & Sugden, 2008; Liu *et al*, 2018). Hence, to further examine the interaction domain of LMP1 responsible for binding to ANT1, we constructed recombinant plasmids encoding a truncated form of Flag‐LMP1 (Fig 2F). In immunoprecipitation assays, we found that only the protein expressed by the LMP1 truncator that retained the transmembrane region was able to bind to ANT1 (Fig 2F), indicating that the transmembrane region is essential for the interaction between LMP1 and ANT1.

The functional unit of ANT1 is a homodimer consisting of two 32 kD proteins, containing six hydrophobic transmembrane sheet layers and three homology domains (Brenner *et al*, 2011; Liu & Chen, 2013). To identify the interaction domain of ANT1 responsible for LMP1 binding, we generated a Myc‐tagged ANT1 deletion construct (Fig 2G). Co‐immunoprecipitation experiments showed that only the constructs of ANT1 retaining the domain2 were able to bind with LMP1 (Fig 2G).

### EBV‐LMP1 induces an ANT1 conformational change

**Figure EV2:**
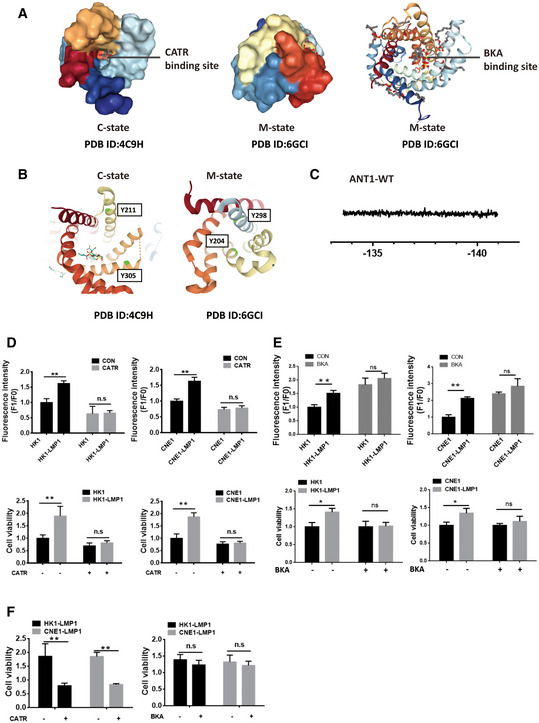
Effect of BKA and CATR inhibitors on nasopharyngeal carcinoma cell function Binding sites of BKA and CATR inhibitors to the ADP/ATP transporter.Spatial position of Y195 and Y290 in two different conformations of ANT1. Y195 (yeast: Y211; *Saccharomyces cerevisiae*: Y204) and Y290 (yeast: Y305; *Saccharomyces cerevisiae*: Y298).19F‐NMR assay of ANT1‐WT as a reference.Effects of CATR on nasopharyngeal carcinoma cell mitochondrial membrane potential and cell viability. “–” represents vehicle treatment only. Data are presented as means ± SEM (paired *t*‐test, *n* = 6, biological replicates per group, **P < 0.01).Effects of BKA on nasopharyngeal carcinoma cell mitochondrial membrane potential and viability. “–” represents vehicle treatment only. Data are presented as means ± SEM (paired *t*‐test, *n* = 6, biological replicates per group, *P < 0.05, **P < 0.01).Cell viability assay in LMP1‐positive NPC cells in CATR/BKA untreated group vs. treated group. “–” represents vehicle treatment only. Data are presented as means ± SEM (paired *t*‐test, *n* = 6, biological replicates per group, **P < 0.01).

**Figure 3:**
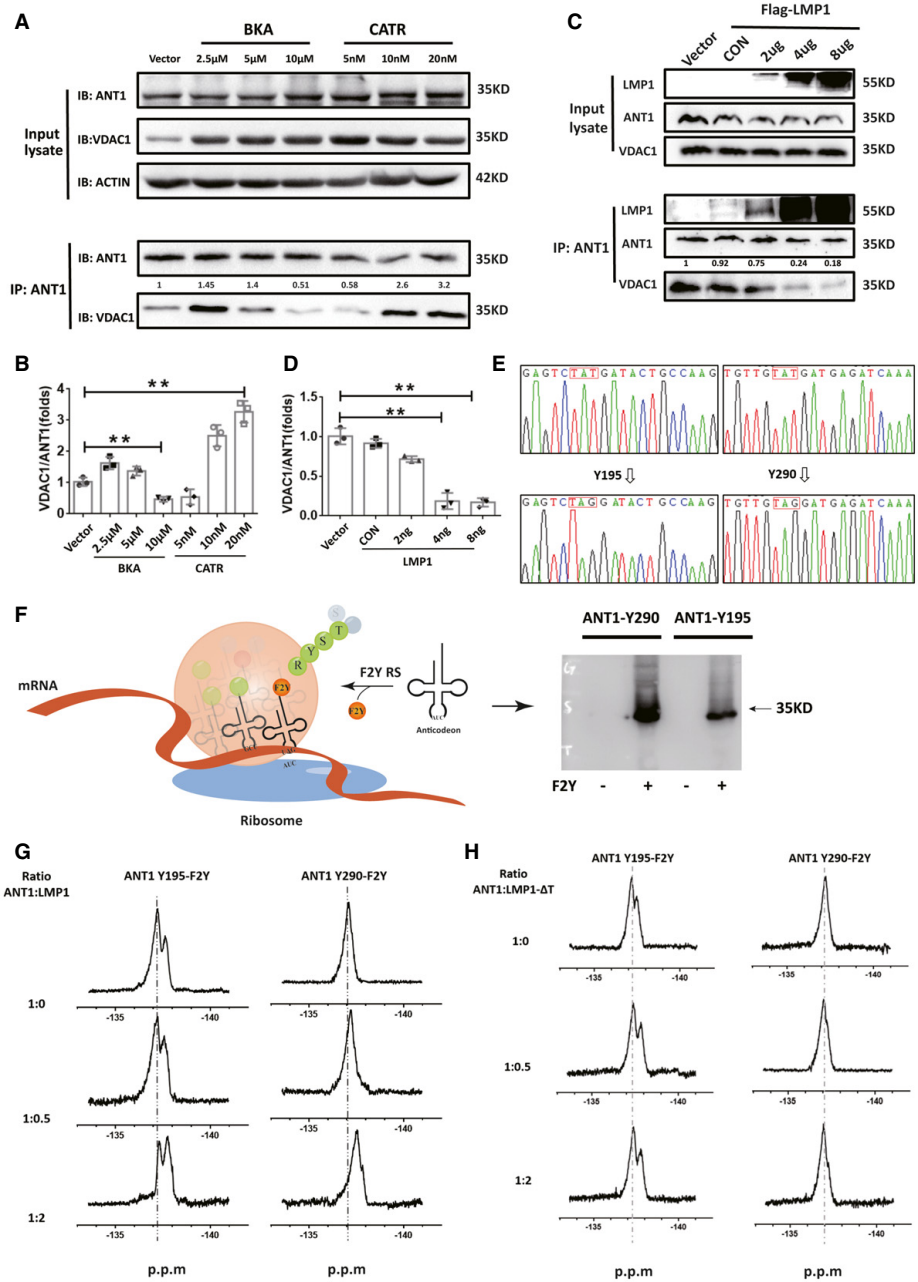
EBV‐LMP1 induces ANT1 conformational changes A, BEffect of inhibitors BKA and CATR on ANT1‐VDAC1 binding in 293T cells. Data are presented as means ± SEM (paired *t*‐test, *n* = 3, biological replicates per group, **P < 0.01).C, DThe effect of different amounts of EBV‐LMP1 on the binding of ANT1‐VDAC1 was detected by CO‐IP. Data are presented as means ± SEM (paired *t*‐test, *n* = 3, biological replicates per group, *P < 0.05, **P < 0.01).ETwo tyrosine sites of ANT1, Y195/Y290 (human origin), were selected and mutated into TAG using a genetic codon extension technique to insert difluorotyrosine into their specific sites to construct two mutant prokaryotic expression vectors of ANT1Y195‐F2Y/ANT1Y290‐F2Y.FSchematic flowchart of the incorporation of F2Y into ANT1.G19F‐NMR spectra of ANT1‐Y195‐F2Y and ANT1‐Y290‐F2Y after titration with different amounts of LMP1. After incubation at room temperature for 30 min, the nuclei were continuously monitored at 500 MZ for 30 min.H19F‐NMR spectra of ANT1‐Y195‐F2Y and ANT1‐Y290‐F2Y after titration with different amounts of LMP1‐ΔT. After incubation at room temperature for 30 min, the nuclei were continuously monitored at 500 MZ for 30 min.

Studies have confirmed that the function of ANT1 is mostly associated with its conformational changes (Ruprecht & Kunji, 2020; Zhao *et al*, 2021); and we confirmed that EBV‐LMP1 and ANT1 are co‐localized in the inner mitochondrial membrane. We next used two specific conformational inhibitors, CATR and BKA, to examine whether the LMP1‐induced changes of mitochondrial membrane potential are associated with ANT1 conformation. We found that CATR inhibited ANT1 at the c‐state, which is one of the necessary conditions for mPTP opening (Novgorodov *et al*, 1992; Ruprecht *et al*, 2019), and BKA maintained ANT1 at m‐state in NPC cells (Fig EV2A). We considered whether EBV‐LMP1 could inhibit ANT1 to maintain it in the m‐state. To test this hypothesis, HK1 cells were treated with different concentrations of BKA or CATR for 24 h, and immunoprecipitation results demonstrated that BKA inhibits the binding of ANT1 to VDAC1, whereas CATR promotes the binding (Fig 3A and B). Next, we transferred LMP1 at increasing amounts into HK1 cells and found that the binding of ANT1 to VDAC1 significantly diminished as LMP1 expression increased (Fig 3C and D), further confirming that LMP1 maintains the conformation of ANT1 in the m‐state and inhibits ANT1‐VDAC1 complex formation.

Due to the 100% natural abundance of fluorine and its sensitivity to the surrounding chemical environment, a wide range of chemical displacements can be seen in the more subtle structural changes and dynamic processes of biomolecules (Yang *et al*, 2015). Here, we inserted difluorotyrosine into the specific site of the ANT1 mutation in the prokaryotic expression system by using genetic codon expansion and then analyzed the LMP1‐induced ANT1 conformational change by using liquid‐phase nuclear magnetic resonance (NMR) (Figs 3E and EV2B; Yang *et al*, 2015).

The relative spatial positions of the two amino acid sites, Y195 and Y290, are considerably changed in the different conformations of ANT1. We selected these two tyrosine sites of ANT1(Y195, Y290) and mutated them into TAG using genetic codon expansion to construct two mutant prokaryotic expression vectors, ANT1Y195‐F2Y/ ANT1Y290‐F2Y (Fig 3F). The ANT1‐WT and two mutant proteins were subjected to nuclear magnetic detection, respectively. No fluorine signal was detected in ANT1‐WT protein by NMR (Fig EV2C). ANT1Y195‐F2Y had a significant fluorine signal (peak at −137.2 p.m). The 19F‐NMR analysis was performed after co‐incubation for 30 min with different concentrations of LMP1. The results showed that the chemical state of fluorine changed with increasing concentrations of LMP1, with a significant chemical shift (peak at −137.9 p.p.m.; Fig 3G). Similar results were observed in the ANT1Y290‐F2Y mutant (Fig 3G). To further verify whether only the binding of LMP1 to ANT1 can induce a conformational change in ANT1, we constructed a prokaryotic expression vector for LMP1 with deletion of the transmembrane domain (LMP1‐ΔT), and after its prokaryotic expression in the strain and purification, different concentrations of LMP1‐ΔT were co‐incubated with two mutants of ANT1 at room temperature for 30 min and then subjected to 19F‐NMR analysis. The state of fluorine did not change as the concentration of LMP1‐ΔT increased (Fig 3H), confirming that the binding of LMP1 to ANT1 is necessary for the conformational change of ANT1.

Next, we examined the Δψm and viability of CNE1/CNE1‐LMP1 and HK1/HK1‐LMP1 cells after treatment with CATR. Both Δψm and viability were significantly higher for CNE1‐LMP1 and HK1‐LMP1 cells compared to CNE1 and HK1 cells (Fig EV2D). No statistical difference was observed in Δψm and cell viability between LMP1‐negative and LMP1‐positive cells after 24 h of CATR treatment. In addition, we examined the Δψm and viability of CNE1/CNE1‐LMP1 and HK1/HK1‐LMP1 cells after treatment with BKA. Both Δψm and viability were significantly higher for CNE1‐LMP1 and HK1‐LMP1 cells compared to CNE1 and HK1 cells (Fig EV2E). No statistical difference was observed in Δψm and cell viability between LMP1‐negative and LMP1‐positive cells after 24 h of BKA treatment, and in the LMP1 positive cells, the cell viability in the CATR untreated group was significantly higher than that in the treated group, and the difference was statistically significant; while in the BKA untreated group, there was no significant difference in cell viability compared with the treated group (Fig EV2F). This result suggests that EBV‐LMP1 maintains the m‐state, inhibits VDAC1‐ANT1 complex formation, increases mitochondrial potential, and promotes NPC cell viability. This suggests that EBV‐LMP1 may modulate Δψm and cell viability by affecting the conformation of ANT1.

### EBV‐LMP1 fixes ANT1 at the m‐state to inhibit ADP/ATP exchange

**Figure 4:**
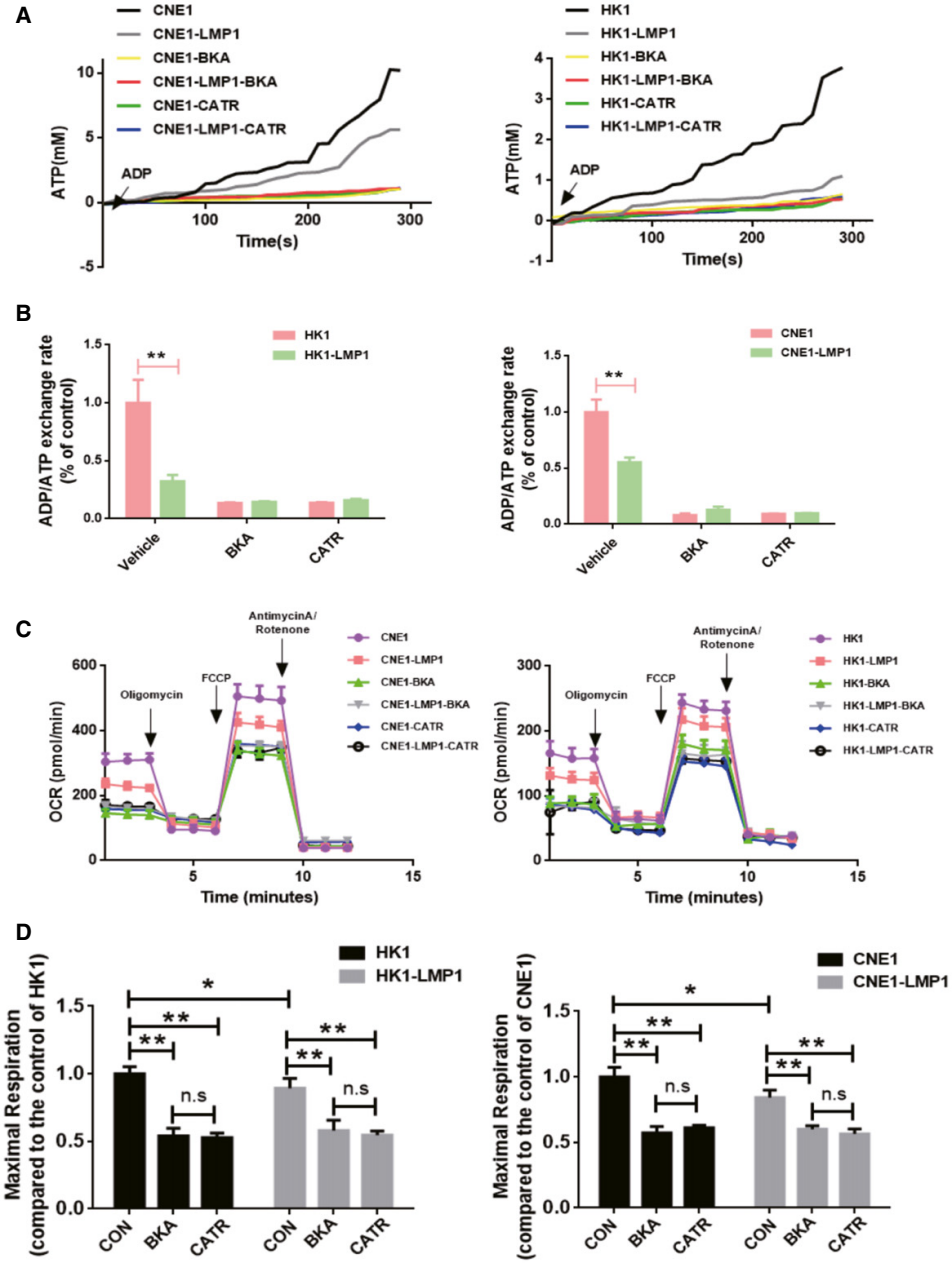
EBV‐LMP1 fixes ANT1 at the m‐state to inhibit ADP/ATP exchange A, BThe ADP/ATP exchange rate in EBV‐LMP1‐positive nasopharyngeal carcinoma cells is decreased. The fluorescence value of Magnesium Green™ (1 μM) was measured at 505 nm (Ex)/535 nm (Em) by enzymography (A), and the ADP/ATP exchange rate was calculated after conversion to ATP (B). Data are presented as means ± SEM (paired *t*‐test, *n* = 5, biological replicates per group, **P < 0.01).CLMP1‐negative or LMP1‐positive NPC cells were treated with BKA (1 μM) or CATR (5 μM) for 24 h, mitochondrial OCR assessed with the XF24 extracellular flux analyzer (Seahorse) under sequential treatment with oligomycin, FCCP, and antimycin A. Data are presented as means ± SEM (*n* = 6, biological replicates per group).DOxidative phosphorylation‐related metrics based on OCR data. Data are presented as means ± SEM (paired *t*‐test, *n* = 6, biological replicates per group, *P < 0.05,**P < 0.01).

The primary function of ANT1 is the exchange of cytosolic ADP and intramitochondrial ATP. We next analyzed the effects of EBV‐LMP1 on the mitochondrial ADP/ATP exchange function. ADP transported by ANT during mitochondrial ATP generation couples with H^+^ and phosphate delivered in the electron respiratory chain to form ATP (Bertholet *et al*, 2019; Ruprecht & Kunji, 2020). We added ADP and Magnesium Green™ to two cell sets, CNE1/CNE1‐LMP1 and HK1/HK1‐LMP1, after cell permeabilization using Digitonin to detect changes in magnesium ion concentration over time, and then obtained absolute values of ADP‐ATP exchange rate (Table 1) according to the method described by Kawamata (Kawamata *et al*, 2010; Zhang *et al*, 2017). The ADP/ATP exchange rate of CNE1‐LMP1/HK1‐LMP1cells was significantly reduced compared with CNE1/HK1 cells (Fig 4A and B). However, in the BKA and CATR groups, the ADP/ATP exchange rates were inhibited and no significant difference was observed (Fig 4A and B).

**Table 1:** ADP/ATP exchange rate in NPC cells (expressed as nmol/s).

Groups	Vehicle	BKA	CATR
CNE1	5.3 ± 2.3	0.62 ± 0.02	0.72 ± 0.01
CNE1‐LMP1	2.2 ± 0.92	0.73 ± 0.04	0.86 ± 0.03
HK1	6.8 ± 1.2	0.61 ± 0.03	0.65 ± 0.01
HK1‐LMP1	3.7 ± 0.98	0.74 ± 0.04	0.60 ± 0.02

Data were expressed as mean ± SD, *n* = 5.

Next, we analyzed the mitochondrial stress status of NPC cells by using the Seahorse XF and found that the oxidative phosphorylation level of HK1‐LMP1 and CNE1‐LMP1 cells was significantly lower than that of HK1 and CNE1 cells (Fig 4C). The levels of oxidative phosphorylation (maximal mitochondrial respiration level) in NPC cells were significantly inhibited by the addition of either BKA or CATR treatment, but no significant differences were observed (Fig 4D). These results showed that mitochondrial ADP/ATP exchange and oxidative phosphorylation levels are suppressed in EBV‐LMP1 cells and the process is closely related to ANT1 conformation‐based functional restrictions.

### CATR inhibits ANT1 in c‐state and attenuates EBV‐LMP1‐induced cisplatin resistance *in vitro*

**Figure 5:**
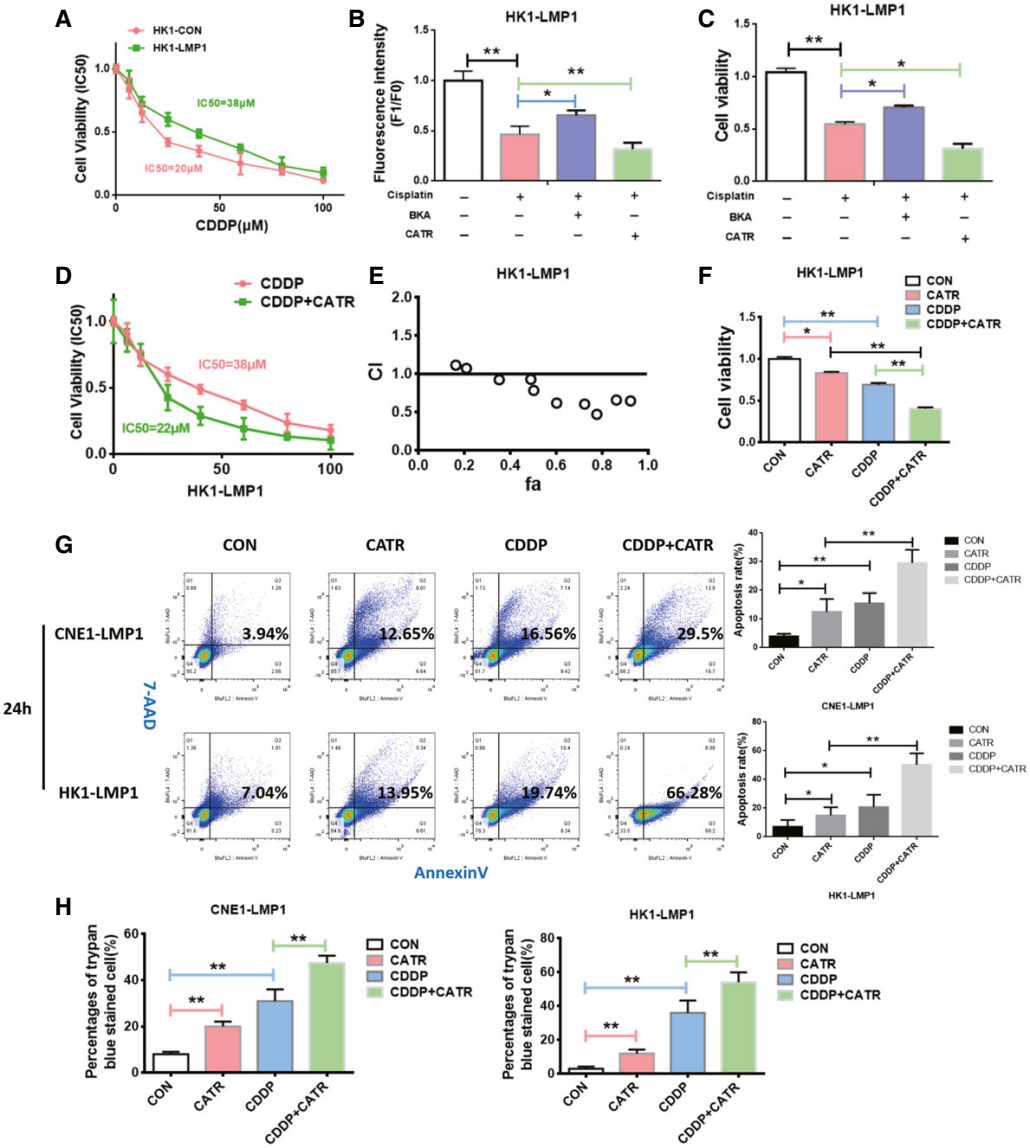
CATR enhances the sensitivity of NPC to cisplatin *in vitro* AHK1‐LMP1 was treated with increasing concentrations of cisplatin for 24 h. Cell viability was determined by MTS assay. Data are presented as means ± SEM (*n* = 6, biological replicates per group).B, CChanges in mitochondrial membrane potential and viability of HK1‐LMP1 cells were assessed 24 h after treatment with cisplatin (20 μM) alone or a cisplatin (20 μM) combination with BKA (1 μM)/CATR (1 μM). Mitochondrial membrane potential of cells was analyzed by using flow cytometry (B), and cell viability was measured by a CCK‐8 assay (C). Data are presented as means ± SEM (paired *t*‐test, *n* = 6, biological replicates per group, *P < 0.05, **P < 0.01).DThe IC_50_ value of the combination of CATR and cisplatin in CNE1‐LMP1 cells was changed. Data are presented as means ± SEM (*n* = 6, biological replicates per group).EThe combination index of cisplatin and CATR in HK1‐LMP1 cells (note: CI < 1 indicates synergism, CI = 1 indicates an additive effect, and CI > 1 indicates antagonism).FCCK‐8 analysis of viability in HK1‐LMP1 cells treated for 24 h with CATR (1 μM) alone, cisplatin (20 μM) alone, or cisplatin (20 μM) combined with CATR (1 μM). Data are presented as means ± SEM (paired *t*‐test, *n* = 6, biological replicates per group, *P < 0.05, **P < 0.01).GFlow cytometry analysis of death of NPC cells treated for 24 h with CATR (1 μM) alone, cisplatin (20 μM) alone, or cisplatin (20 μM) combined with CATR (1 μM). Data are presented as means ± SEM (paired *t*‐test, *n* = 5, biological replicates per group, *P < 0.05, **P < 0.01).HTrypan blue staining was used to detect death of NPC cells treated for 24 h with vehicle, cisplatin, CATR, or a combination of cisplatin and CATR (1 μM). Data are presented as means ± SEM (paired *t*‐test, *n* = 6, biological replicates per group, **P < 0.01).

**Figure EV3:**
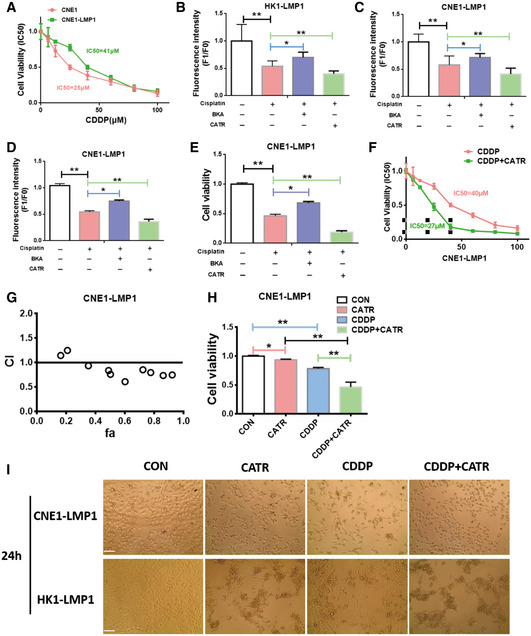
CATR enhances the sensitivity of NPC cells to cisplatin ACNE1 and CNE1‐LMP1 cells were treated for 24 h with increasing concentrations of cisplatin. Cell viability was determined by MTS assay. Data are presented as means ± SEM (*n* = 6, biological replicates per group).B, CLMP1‐positive NPC cells were treated with cisplatin for 24 h and BKA (1 μM)/CATR was added for immediate detection of mitochondrial membrane potential. Data are presented as means ± SEM (paired *t*‐test, *n* = 6, biological replicates per group, *P < 0.05, **P < 0.01).D, EChanges in mitochondrial membrane potential and viability of and CNE1‐LMP1 cells treated for 24 h with cisplatin (20 μM) alone or cisplatin (20 μM) combined with BKA (1 μM)/CATR (1 μM). Mitochondrial membrane potential of cells was analyzed by using flow cytometry (B), and viability was measured by the CCK‐8 assay (C). Data are presented as means ± SEM (paired *t*‐test, *n* = 6, biological replicates per group, *P < 0.05, **P < 0.01).FThe IC_50_ value of the combination of CATR and cisplatin in CNE1‐LMP1 was changed. Data are presented as means ± SEM (*n* = 6, biological replicates per group).GThe combination index of cisplatin and CATR in CNE1‐LMP1 cells (note: CI < 1 indicates synergism; CI = 1 indicates an additive effect; and CI > 1 indicates antagonism).HCCK‐8 analysis of viability in CNE1‐LMP1 cells treated for 24 h with CATR (1 μM) alone, cisplatin (20 μM) alone, or cisplatin (20 μM) combined with CATR (1 μM). Data are presented as means ± SEM (paired *t*‐test, *n* = 6, biological replicates per group, *P < 0.05, **P < 0.01).IMorphology of NPC cells treated for 24 h with vehicle, cisplatin, CATR, or a combination of cisplatin and CATR (scale bar, 100 μm).

Here, it was found that the IC50 of cisplatin in LMP1‐negative cells (HK1 20 μM, CNE1 25 μM) was dramatically lower than that of EBV‐LMP1‐positive cells (HK1‐LMP1 38 μM, CNE1‐LMP1 41 μM) (Figs 5A and EV3A). Therefore, it was determined whether this chemoresistance was related to EBV‐LMP1‐mediated maintenance of the m‐state ANT1 conformation. We detected Δψm and viability of EBV‐LMP1‐positive NPC cells treated with cisplatin alone and cisplatin in combination with BKA or CATR by using flow cytometry and CCK‐8 assay, respectively. It was shown that in LMP1‐positive cells, the combination of CATR with cisplatin significantly reduced Δψm and viability compared to cisplatin alone. (Figs 5B and C, and EV3D and E). In addition, we detected the mitochondrial membrane potential by adding BKA and CATR immediately after cisplatin treatment of LMP1‐positive NPC cells for 24 h (Fig EV3B and C), and the results were similar to those after 24 h of BKA and CATR treatment. Additionally, we compared the IC_50_ values of cisplatin alone and cisplatin in combination with CATR in HK‐LMP1 and CNE‐LMP1 cells and found that the IC_50_ was significantly lower in the combined group (HK1‐LMP1 22 μM, CNE1‐LMP1 27 μM), suggesting that cisplatin resistance in EBV‐LMP1 cells is closely related to the conformational changes in ANT1, whereas CATR can increase cisplatin‐induced cell death (Figs 5D and EV3F). To determine whether the effect of combining cisplatin and CATR was synergistic (greater than the sum of the group effects), combination indexes (CI) were calculated from the dose–response data. HK1‐LMP1 and CNE1‐LMP1 cells treated with the combination of cisplatin and CATR had CI values < 1 indicating a synergistic effect (Figs 5E and EV3G).

In order to verify the biological effect of CATR and cisplatin in EBV‐LMP1 NPC cells, cells were divided into a control group, CATR treatment‐only group, CDDP treatment (cisplatin treatment group) only group, and a combination of CDDP‐and CATR‐treated group, respectively. We observed that the cells in the CATR combined with cisplatin group grew more slowly and had a more rounded and brighter morphology than those in the cisplatin treatment‐only group (Fig EV3I). Cell proliferation was detected by CCK‐8, and viability was found to be significantly lower in the cisplatin and CATR combined group compared to the other three groups (Figs 5F and EV3H).

Analysis by Annexin V (PE) and 7‐AAD staining revealed significantly higher mortality in the CNE1‐LMP1 and HK1‐LMP1 combination groups (29.5% and 66.28%) compared to the cisplatin only group (16.56% and 19.74%) and CATR only group (12.65% and 13.95%; Fig 5G). The staining results were divided into two categories of apoptosis (PE^+^/7‐AAD^−^) and necrosis (7‐AAD^+^), and we found that both apoptosis and necrosis were present in the combined group, and apoptosis was predominant (Fig 5G). Finally, we observed cell death by trypan blue staining and found that cell death was significantly higher in the cisplatin and CATR combination group compared with the other three groups (Fig 5H).

### CATR enhances the sensitivity of EBV‐LMP1‐positive cells to cisplatin *in vivo*

**Figure 6:**
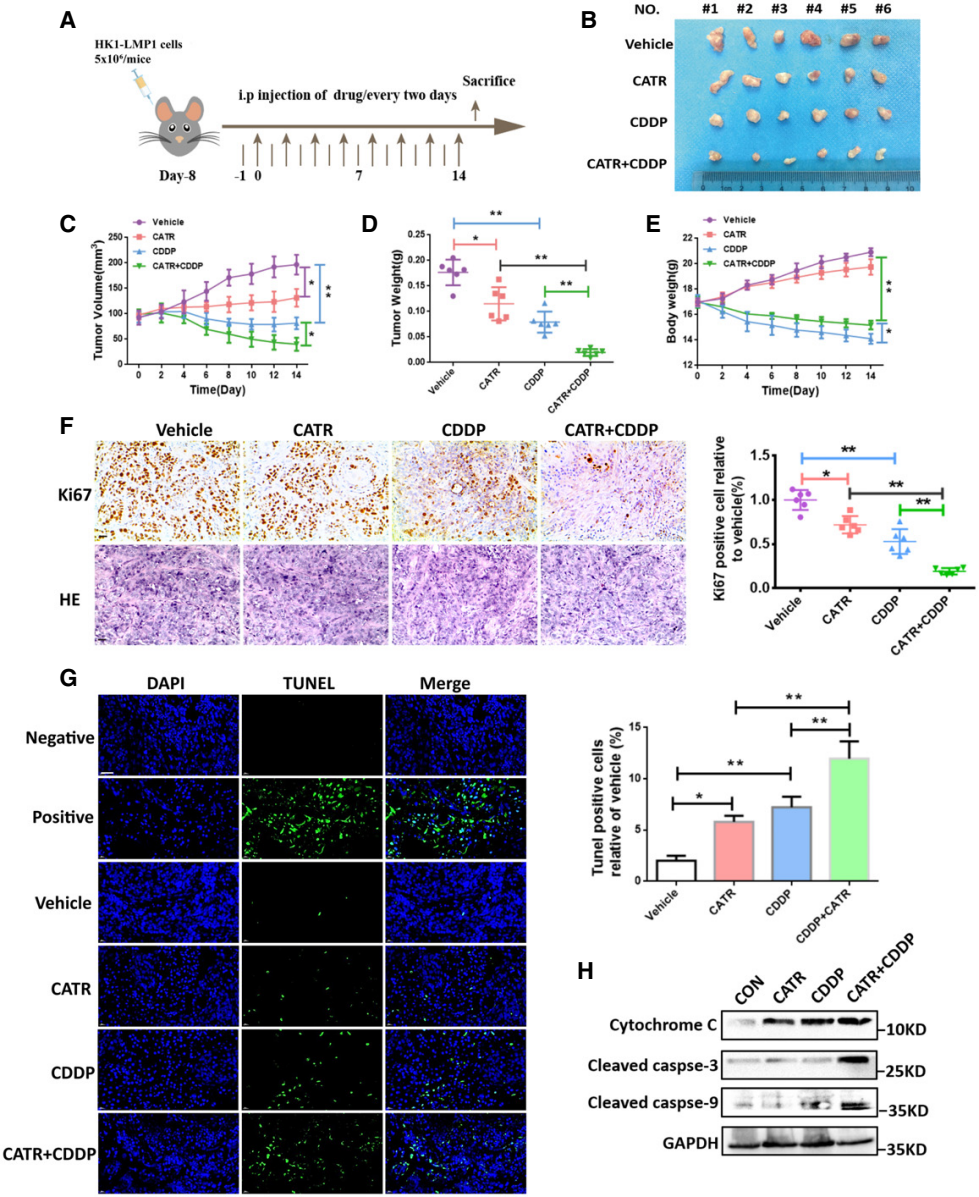
CATR enhances the sensitivity of EBV‐LMP1‐positive cells to cisplatin *in vivo* AThe overall schematic diagram of the study design. The nasopharyngeal carcinoma xenograft model was established using HK1‐LMP1 cells.BRepresentative images of xenograft tumors from different treatment groups.C, DThe tumor volume (C) and tumor weight (D) of HK1‐LMP1‐derived xenograft tumors with various treatments. Data are presented as means ± SEM (paired *t*‐test, *n* = 6, biological replicates per group, *P < 0.05, **P < 0.01).EThe mouse weight curve of the different treatment groups in a HK1‐LMP1 cell xenograft model. Data are presented as means ± SEM (paired *t*‐test, *n* = 6, biological replicates per group, *P < 0.05, **P < 0.01).FImmunohistochemistry examination of the protein levels of Ki67 (scale bar, 50 μm). Data are presented as means ± SEM (paired *t*‐test, *n* = 6, biological replicates per group, *P < 0.05, **P < 0.01).GApoptosis was analyzed by TUNEL assay (scale bar, 50 μm). Data are presented as means ± SEM (paired *t*‐test, *n* = 6, biological replicates per group, *P < 0.05, **P < 0.01).HApoptosis was analyzed by Western blotting. Source data are available online for this figure.

The effect of cisplatin combined with CATR was examined in a xenograft mouse model to further determine whether CATR sensitizes NPC to cisplatin by modulating the conformational changes of ANT1 *in vivo* (Fig 6A). We found a significant reduction in tumor volume (Fig 6B and C) and tumor weight (Fig 6D) in the CATR and cisplatin combination groups compared to the vector group, CATR group, and cisplatin alone.

*In vivo* experiments showed that Ki67 expression was significantly reduced in the CATR and cisplatin combined treatment group (Fig 6F), and the apoptosis rate was significantly increased as determined by a TUNEL detection kit (Fig 6G). We observed significantly enhanced levels of caspase‐3, caspase‐9, and cytochrome c in the combined CATR and cisplatin group compared to the vector or cisplatin‐only group (Fig 6H). In addition, it was observed that the body weight (B.W.) of mice decreased significantly in the cisplatin‐treated group, but not in the CATR‐treated group (Fig 6E). Interference with the ANT1 inhibitor CATR increases the chemosensitivity of tumor cells to cisplatin. Together, these results suggest that ANT1 is a potential target for reducing the proliferation and survival of NPC cells.

## Discussion

The mitochondrial permeability transition is a phenomenon of a significant increase in mitochondrial inner membrane permeability, which is mainly caused by the opening of the mPTP (Zorova *et al*, 2018; Brustovetsky, 2020). Prolonged and massive opening of mPTP leads to mitochondrial swelling and the release of associated factors such as cytochrome C and AIF, which eventually leads to cell death (Zorov *et al*, 2014; Zorova *et al*, 2018; Bock & Tait, 2020; Zhao *et al*, 2021). We found that EBV‐LMP1 localizes to the inner mitochondrial membrane and reduces the opening of mPTP by inhibiting the formation of the ANT1‐VDAC1 complex, leading to an increase in the Δψm. EBV‐LMP1 was found to localize to the endoplasmic reticulum membrane to induce unfolded protein responses and activate multiple signaling pathways that regulate the physiology of EBV‐infected B cells in unexpected ways (Lee & Sugden, 2008; Meckes *et al*, 2013; Wang *et al*, 2014). Thus, mitochondrial localization of LMP1 may represent a novel molecular event that promotes the activity of NPC cells.

Conformational changes of proteins are known to be closely related to their functions; however, the conformational changes of proteins in carcinogenesis are not yet well explained. The function of ANT1 is closely related to its conformational changes. For example, the ADP/ATP cycle of ANT1 regulates energy metabolism by switching between the c‐state and m‐state, the c‐state of ANT1 promotes mPTP opening to induce cell death, and recent resolution of the m‐state conformation has further refined the mechanism of ADP/ATP transport (Novgorodov *et al*, 1992; Ruprecht *et al*, 2019). In this study, the transmembrane structural domain of EBV‐LMP1 was found to bind directly to structural domain 2 of ANT1. Using conformational inhibitors of ANT1 and 19F‐NMR, we further confirmed that LMP1 fixed the conformation of ANT1 in m‐state, thereby inhibiting the opening of mPTP and improving cell viability, and that LMP1 binding to ANT1 is required for this process. This involved the cytoprotective role of EBV infection inducing the mitochondrial stress microenvironment. In addition, EBV‐LMP1 cells had a restricted mitochondrial ADP/ATP exchange and reduced levels of cellular oxidative phosphorylation were found by using hippocampal XF mitochondrial stress analysis, indicating that LMP1 reduced ATP production through mitochondria. A new perspective confirms that conformational changes in ANT1 due to LMP1 affect the metabolic profile of NPC cells and inhibit the level of oxidative phosphorylation in mitochondria, a process that has been shown to be a key factor in the poor prognosis of NPC (Xiao *et al*, 2014; Lu *et al*, 2016; Luo *et al*, 2018).

The mPTP is closely associated with chemoresistance, and its key components ANT1/2/3 and VDAC1 play important roles in mitochondrial cisplatin resistance (Tajeddine *et al*, 2008; Sancho‐Martínez *et al*, 2012; Galluzzi *et al*, 2014; Briston *et al*, 2019). VDAC‐ANT1 complex formation is an important molecular event for mPTP opening and plays a key role in drug‐induced mitochondrial death (Wang *et al*, 2014). CATR, an ANT1‐specific inhibitor that maintains ANT1 in c‐state to promote VDAC‐ANT1 complex formation, has been shown to play a protective role in hepatic steatosis and insulin resistance in mice (Cho *et al*, 2017). Although no anti‐tumor applications of CATR have been reported, it has potential as a molecular inhibitor important for maintaining mitochondrial energy metabolism and membrane potential. We demonstrate that CATR enhances the *ex vivo* chemosensitivity of NPC cells to cisplatin by inhibiting ANT1, which contributes to mPTP opening and cell death. These data suggest that ANT1 is a novel therapeutic target for overcoming cisplatin resistance.

**Figure 7:**
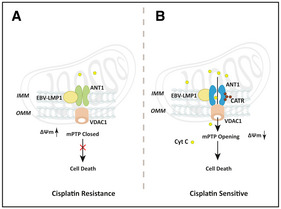
A schematic to illustrate that the EBV‐LMP1 affects chemotherapy by regulating the conformation of ANT1 EBV‐LMP1 inhibits the ANT1 conformation during cisplatin chemotherapy alone to keep it in the m‐state and prevent the opening of mPTP to resist cell death (A). CATR, a conformational inhibitor of ANT1, used in combination with cisplatin increases the chemosensitivity in EBV‐LMP1‐positive NPC (B).

In conclusion, we report that the c‐state of ANT1 is beneficial to the formation of the ANT1‐VDAC1 complex, which induces the opening of mPTP and the decrease of membrane potential. EBV‐LMP1 was first discovered to localize to the mitochondrial inner membrane and inhibit mPTP opening by binding to ANT1 fixed to m‐state, thereby favoring NPC cell survival and chemoresistance. CATR combined with cisplatin improves chemotherapy sensitivity of EBV‐LMP1‐positive cells (Fig 7). This finding confirms that ANT1 may be a key target for improving the sensitivity of NPC to cisplatin chemotherapy in the future.

## Materials and Methods

### Reagents and antibodies

Reagents were purchased as follows: bongkrekic acid (19079, Cayman, USA), carboxyatractyloside (19079, MCE, China), cisplatin (HY‐17394, MCE, China), calcein‐AM (17783, Sigma‐Aldrich, USA), CoCl_2_ (V900021, Sigma‐Aldrich, USA), calcimycin (A23187, HY‐N6687, MCE, China), Magnesium green™ (M3733, Invitrogen, USA), STMRM, (T668, Invitrogen, USA), ATP Determination Kit (A22066, Invitrogen, USA), CCK‐8 (CK04, Dojindo, Japan), GST tag Purification Kit (P2262, Beyotime, China), MultiS Fast Mutagenesis Kit (C214, Vazym, China), 3,5‐difluoro‐L‐tyrosine (CAS 73246‐30‐7, Yiji Biotechnology, Shanghai, China).

Antibodies used included ANT1 mAb (ab110322, Abcam, UK, 1:1,000), VDAC1 mAb (Ab34726, Abcam, UK, 1:1,000), HSP60 mAb (Sc‐1052, Santa Cruz Biotechnology, USA, 1:1,000), β‐actin mAb (A5441, Sigma, Germany, 1:10,000), cyto C mAb (556433, BD, USA, 1:1,000), TIM23 mAb (11123‐1‐AP, Proteintech, USA, 1:1,000), ANT2 mAb (14671S, CST, USA, 1:2,000), VDAC2 mAb (11663‐1‐AP, Proteintech, USA, 1:1,000), caspase‐9 mAb (9502S, CST, USA, 1:1,000), Flag mAb (F1804, Sigma‐Aldrich, USA, 1:1,000), and cleaved caspase‐3 mAb (9664S, CST, USA, 1:1,000).

### Cell lines and culture

CNE1: Human nasopharyngeal squamous carcinoma cell, EBV antigen negative, purchased from the Cell Resource Center of Central South University; HK1: Human nasopharyngeal carcinoma cell line from nasopharyngeal carcinoma biopsy tissue, EBV antigen negative, gift from Prof Sai‐Wah Tsao, The University of Hong Kong. CNE1‐LMP1 and HK1‐LMP1 are stably LMP1 expressing human nasopharyngeal carcinoma cells and were constructed in our laboratory. NPC cells were cultured in RPMI‐1640 medium (Gibco, USA) with 10% fetal bovine serum (Hyclone) in a 5% CO_2_ incubator at 37°C. The human embryonic kidney cell line HEK293T was cultured in Dulbecco's modified Eagle medium (DMEM, Hyclone) containing 10% FBS (Hyclone) in a 5% CO_2_ incubator at 37°C. All cells were tested regularly for mycoplasma.

### Mitochondrial membrane potential (Δψm)

The ΔΨm was measured by using two different dyes and FACScan flow cytometer or confocal microscopy. For FACScan flow cytometer detection, the cells were stained with JC‐1 (10 μg/ml, 37°C) for 15 min under standard growth conditions, washed with PBS, and analyzed by using a EPICS XL flow cytometer. For detection by confocal microscopy, cells were seeded in a glass culture dish, stained with TMRM (200 nM, 37°C) for 20 min, and washed three times with PBS. The fluorescence was examined by microscopy (LSM 510 META, Germany) and analyzed using ImageJ software (NIH).

### Mitochondrial swelling assays

As described by Chen (Zhang *et al*, 2017) one or 2 mg of isolated mitochondria was loaded into a cuvette along with 250 nM calcium green‐5N (Invitrogen), 7 mM pyruvate (Sigma‐Aldrich), and 1 mM malate (Sigma‐Aldrich) and brought up to 1 ml with KCl buffer. Mitochondria were then pulsed with sequential additions of CaCl_2_ (40 or 800 µM) until mPTP opening occurred or until the mitochondria reached a CaCl_2_ saturation point and could no longer take up more Ca^2+^. Ca^2+^‐induced mitochondrial swelling was assessed by the decrease in absorbance at 540 nm absorbance for 600 s and the decrease in curve represented the degree of mitochondrial swelling. The curves represent typical recordings from experiments of at least three different mitochondrial preparations.

### Determination of mPTP opening

Cells cultured in 24‐well plate were collected and incubated with calcein‐AM (2 μM) in a medium containing 120 mM NaCl, 5.0 mM KCl, 2.0 mM CaCl_2_, 20 mM HEPES, and 15 mM glucose (pH 7.4) at 37°C for 30 min, which causes penetration into the cytoplasm and mitochondria. The fluorescence from cytosolic dye was quenched for another 30 min by the addition of CoCl_2_ (1 mM). After washing cells three times, half of the cells were left as is, and the other half were treated for another 30 min with 500 μM CaCl_2_, Ca^2+^ ionophore calcimycin (5 μM), CATR (1 μM), or BKA (5 μM) to trigger or inhibit mPTP opening. Then, the calcein fluorescence was detected by the Varioskan flash instrument (Thermo Scientific, USA) and the decreased percentage of initial calcein fluorescence could be accepted as the mPTP opening level.

### Plasmids and siRNA

The siRNAs were designed and synthesized by RIBOBIO (Guangzhou, China) (Table EV1). The full‐length expression vectors for Myc‐ANT1, Flag‐LMP1, and Myc‐VDAC1 and the deletion constructs of ANT1 and LMP1 were designed and synthesized by our laboratory. All DNA sequencing of the plasmids was verified by TSINGKE (Beijing, China; Table EV2). Plasmid transfections were performed by using Lipofectamine 2000 (Invitrogen) according to the manufacturer’s protocol.

### Mitochondrial isolation

The mitochondrial extract was prepared in MSHE + BSA buffer (210 mM mannitol, 70 mM sucrose, 5.0 mM HEPES, 1.0 mM EGTA, 0.5% (w/v) fatty acid‐free bovine serum albumin (BSA), and complete protease inhibitor cocktail, pH 7.2). Each sample collected required 2–4 × 10^7^ cells, which were suspended in 1 ml MSHE + BSA buffer, gently mixed, and placed on ice for 5 min. Subsequently, the supernatant fraction was collected by centrifugation following the homogenization of mitochondria. The supernatant fraction was obtained by high‐speed centrifugation (11,304 *g* for 30 min at 4°C) from isolated mitochondria and contained cytoplasmic proteins.

### Preparation of mitochondrial fractions

Mitochondria were incubated for 30 min on ice with 20 or 40 μg of proteinase K (Sigma‐Aldrich). Incubation was stopped with 1 mM PMSF, and the samples were analyzed by Western blotting. Mitochondrial samples were disrupted in 0.1 M Na_2_CO_3_ (pH 11.5) on ice for 30 min with occasional vortexing. The membranes were isolated by centrifugation at 100,000 *g* for 30 min at 4°C and analyzed by Western blotting.

### Western blot analysis

Cells were harvested and disrupted in IP lysis buffer (25 mM Tris–HCl pH 7.4, 150 mM NaCl, 1% NP40, 1 mM EDTA, 5% glycerol; Thermo Scientific, MA, USA). Extracted proteins were separated by SDS–‐PAGE and transferred onto nylon membranes. Binding of primary antibodies was detected using peroxidase‐conjugated secondary antibodies. Visualization was performed by using the ChemiDoc XRS system with Image Lab software (Bio‐Rad, CA, USA).

### Confocal microscopy

Cells were washed twice with PBS and fixed in 4% para‐formaldehyde. The cells were incubated with an appropriate primary antibody for 3 h at 37°C, washed with PBS, and incubated with FITC‐ or Texas Red‐conjugated secondary antibodies (Life Technologies) for another 3 h. The cells were stained with 4′,6‐diamidino‐2‐phenylindole (DAPI, 50 μg/ml) for 5 min for the detection of nuclei by TCS SPISE confocal microscopy (Leica).

### Co‐immunoprecipitation

Cells were disrupted with IP lysis buffer containing protease inhibitor cocktails (Roche Diagnostics, Basel, Switzerland). Protein aliquots (500 μg) were pre‐cleared by incubation with 20 μl of Dynabeads protein A (Invitrogen, MA, USA) for 1 h at 4°C. The pre‐cleared samples were incubated with antibody (2 μg/sample) overnight at 4°C. Then, 20 μl Dynabeads protein A were added to samples and incubated for 2 h at 4°C. The beads were washed 3 times with cold lysis buffer, then boiled and analyzed by Western blotting.

### Proximity ligation assay (PLA)

The DuoLink® *In Situ* Red Starter Kit Mouse/Rabbit (DUO92101) purchased from Sigma‐Aldrich was used for this experiment. Cells seeded in eight‐well chamber slides were washed with PBS, fixed in 4% para‐formaldehyde for 30 min, and then permeabilized in 0.1% Triton X‐100 for 20 min. Slides were then blocked with Duolink blocking solution in a pre‐heated humidity chamber for 30 min at 37°C and incubated with the primary antibody to detect Myc/Flag (or ANT1/LMP1/VDAC1) overnight at 4°C. On the following day, slides were incubated with the PLA probes diluted 1:5 in antibody diluents in a pre‐heated humidified chamber for 1 h at 37°C. Subsequent hybridization, ligation, amplification, and detection were performed according to the manufacturer’s protocol. Fluorescence images were acquired using a Leica TCS SP5 confocal microscope.

### Expression and purification of native ANT1

The PGEX‐4T‐1‐ANT1 plasmid was transformed into BL21 *E*. *coli*. The transformed BL21 cells were cultured and induced with 0.3 mM isopropyl‐b‐D‐thiogalactoside (IPTG) until an OD600 nm of 0.8 was reached. After growing for 6 h (37°C), the cells were harvested, re‐suspended in lysis buffer (20 mM Tris–HCl pH 8.0, 150 mM NaCl), and disrupted by sonication. The bacterial lysate was then centrifuged and the supernatant fraction was purified following the manufacturer’s instructions (Beyotime, P2262).

### Expression and purification of F2Y‐incorporated proteins

The genetic codon extension technique was pioneered by Schultz *et al*. (Wang *et al*, 2001) by using orthologous amber repressive tRNAs with corresponding tRNA synthetases to insert unnatural amino acids at specific sites in the target protein. The specific site of ANT1 (Y195/Y290) was mutated to TAG and difluorotyrosine was inserted into the specific mutated site of ANT1 by using the codon extension technique (Wang *et al*, 2001; Yang *et al*, 2015). For the expression of ANT1 F2Y proteins, pEVOL‐F2YRS (Prof. Jiangyun Wang from the Chinese Academy of Sciences gifted the plasmid) was co‐transformed with different PGEX‐4T‐1‐ANT1 TAG mutations into BL21 (DE3). A single colony was grown overnight at 37°C in Luria–Bertani (LB) medium. Five liters of the transformed cells were then induced with 0.3 mM IPTG and 0.02% L‐arabinose at an OD600 nm of 1.0 in the presence of 0.5 mM F2Y. After growing 6 h (37°C), the cells were harvested and resuspended in buffer containing 20 mM Tris–HCl pH 8.0 and 150 mM NaCl. The bacterial lysate was next centrifuged, and the supernatant fraction was purified by following the manufacturer’s instructions (Beyotime, P2262).

### 19F‐NMR

Liquid‐phase NMR spectroscopy is a powerful tool for studying the structure and dynamics of biomolecules under physiological conditions, and the insertion of non‐natural amino acids into specific sites of proteins allows the dynamic study of conformational changes and structural information of proteins using NMR techniques. ANT1‐Y195‐F2Y or ANT1‐Y290‐F2Y (50 mg) proteins were incubated at different ratios of LMP1/LMP1‐ΔT (1 :0, 1:0.5 and 1:2) were mixed and incubated in binding buffer for 30 min at room temperature, followed by liquid‐phase 19F‐NMR detection. All NMR data were collected using an Agilent OD2600 spectrometer equipped with a 5‐mm broadband probe. Data were processed using a Lorentz line broadening of 10 Hz and referenced to an internal TFA standard (−76.5 ppm.). All spectra were recorded at 25°C.

### Cell viability and death assays

After the cells are plated in the 96‐well plate, remove the medium from the 96‐well plate with a pipette, take the mixture of complete medium and CCK‐8 solution (10:1), add 100 μl of the mixture to each well, shake gently, incubate at 37°C in 5% CO_2_ incubator for 30 min, take out the 96‐well plate, shake it well, and read the absorbance value at 450 nm on the enzyme standard meter. The absorbance value of each well was read at 450 nm wavelength of the microplate reader.

For the trypan blue test, the cell suspension and 0.4% trypan blue were mixed 9:1, and the mortality of the cells was observed and counted under the microscope within 3 min.

### Combination index

The combination index (CI) was calculated by using the Chou–Talalay equation (Chou & Talalay, 1983; Tsai *et al*, 2018). The general equation for the classic isobologram (CI = 1) is given by: CI = (D)1/(Dx)1 + (D)2/(Dx)2 (A) where (Dx)1 and (Dx)2 in the denominators are the doses (or concentrations) of D1 (drug #1) and D2 (drug #2) alone that gives x% inhibition, whereas (D)1 and (D)2 in the numerators are the doses of D1 and D2 in combination that also inhibits x% (isoeffective). CI < 1 indicates synergism, CI = 1 indicates an additive effect, and CI > 1 indicates antagonism.

### Analysis of the ATP‐ADP exchange rate

The ATP‐ADP exchange rate was measured as previously described (Kawamata *et al*, 2010; Zhang *et al*, 2017). Cells were cultured in 12‐well plates, suspended in buffer (8 mM KCl, 110 mM K‐gluconate, 10 mM NaCl, 10 mM Hepes, 10 mM KH_2_PO_4_, 5 μM EGTA, 10 mM mannitol, 25 μM AP5A, 5 mM NaF, 0.2 mM BeSO_4_, 30 μM Na_3_VO_4_, 5 μM EDTA and 0.5 mg/ml bovine serum albumin (fatty acid‐free, pH 7.25) and treated with 50 μM digitonin. After the addition of ADP (2 mM) and Magnesium Green™ (1 μM, a fluorescent magnesium indicator), fluorescence was recorded by the Varioskan flash instrument (Thermo Scientific, USA) using 505 and 535 nm excitation and emission wavelengths, respectively. The rate of ATP appearing in the medium was calculated from the measured rate of change in free extra‐mitochondrial Mg^2+^.

The minimum fluorescence value (*F*_min_) was recorded by adding EDTA (5 mM), and the maximum fluorescence value (*F*_min_) was recorded by adding MgCl_2_ (10 mM) to the wells to be measured, respectively. The Mg^2+^ concentration was calculated according to the following equation.

$$\left[{\text{Mg}}^{2+}\right]\;f=\left(K_{d}\left(F‐F_{\text{min}}\right)/\left(F_{\text{max}}‐F\right)\right)\;‐0.068\;\text{mM},\;\left(K_{d}=0.\text{9 mM}\right).$$

The concentration of ATP with time (ATPt) was calculated from the Mg^2+^ concentration according to the method as previously described (Kawamata *et al*, 2010; Zhang *et al*, 2017), and then, the slope of ATPt with time was calculated using SPSS data software, which is the ATP/ADP exchange rate.

### Mitochondrial stress analysis

Inoculate 8 × 10^3^ cells on XF96 cell culture microplates, add pre‐warmed XF hydration solution to XF96 probe plate for probe hydration, 200 µl per well, place at 37°C, CO_2_‐free incubator overnight. Then, 180 µl of XF reagent medium was added and placed in a CO_2_‐free incubator at 37°C for 1 h before adding 2 µM oligomycin, 2 µM FCCP, and 0.5 µM rotenone to treat the cells sequentially. The cell plates were placed on the XF96 cell energy metabolism analyzer and quasi‐treated according to the number of cells per group at the end of the process.

### Annexin V staining

Annexin V staining precedes the loss of membrane integrity that characterizes the later stages of cell death, and therefore the vital dye 7‐AAD, in conjunction with annexin V, was used to allow discrimination of early apoptotic cells. Cells were treated with CATR alone (1 µM), cisplatin (20 µM) alone, or cisplatin (20 µM) in combination with CATR (1 µM) for 24 h. Treated cells were harvested and stained with annexin V‐APC (red; BD Pharmingen) at room temperature for 15 min followed by one wash with 1x Annexin V binding buffer. Cells were then incubated with 7‐AAD‐phycoerythrin‐cyanide 7 (PE‐Cy7) at room temperature for 10 min. The percentage of annexin V‐positive cells was determined by using FlowJo software (version 7.2.5).

### TUNEL assay

The ApopTag Peroxidase *In Situ* Apoptosis Detection kit (Chemicon International, Temecula, CA) was used for measuring apoptosis in paraffin‐embedded tumor sections by labeling and detecting DNA strand breaks by using the TUNEL method. Tumor sections (5 µM) were labeled at the free 3’OH DNA termini *in situ* with digoxigenin‐labeled and unlabeled nucleotides added to the DNA by terminal deoxynucleotidyl transferase (TdT). The labeled DNA fragments then bind a digoxigenin antibody conjugated to a peroxidase reporter molecule, which generates a strong stain from chromogenic substrates. Numbers of apoptotic cells were counted in 10 randomly selected high‐power fields. Each slide was examined on at least two separate occasions by two different individuals.

### Xenograft studies

The *in vivo* study was approved by the Medical Ethics Committee (for experimental animals) of the Xiangya Hospital, Central South University. The Department of Zoology of Central South University has a well‐equipped animal room of SPF level, equipped with ultra‐clean bench, anesthesia machine, etc. The purchase and feeding of experimental animals are in accordance with national standards, ensuring the smooth conduct of animal experiments.

Female BALB/c‐nude mice (5–6 weeks) were purchased from SLAC Laboratory Animal Co. Ltd. (Changsha, China). All mice were subcutaneously inoculated with HK1‐LMP1 cells (5 × 10^6^ cells/mouse). When the tumor volume reached 100 mm^3^, the mice were divided randomly into six groups (*n* = 6; saline‐treated control, carboxyatractylosid‐treated, cisplatin‐treated, and cisplatin and carboxyatractylosid‐treated). Single‐drug treatment with carboxyatractylosid (1 mg/kg) or cisplatin (4 mg/kg) was initiated by i.p. injection. The vehicle control was administered in 0.9% saline. Tumor volume and B.W. were recorded every 2 days. The tumor size was calculated as follows: tumor size = ab^2^/2, where a and b are the larger and smaller diameters, respectively. After 15 days of treatment, the mice were euthanized, and tumors were removed and weighed.

### Statistical analysis

All statistical calculations were performed by using the GraphPad Prism 5 software program (GraphPad Software). The experimental data are presented as the mean values ± SEM. The statistical significance of the data was analyzed by using a standard Student’s *t*‐test. A *P*‐value of < 0.05 was deemed statistically significant, and *P* < 0.01 was considered statistically significant. All *P*‐values are provided in the Appendix Table S1.

## Author contributions

Supervision: YC; study concept and design: LZ and YC; drafting of the manuscript: LZ and YC; collection, analysis, or interpretation of data: XD, YL, JH, LX, MT, and AMB; technical or material support: XZ, FS, and WL.

## Conflict of interest

The authors declare that they have no conflict of interest.

## Supporting information



Appendix

Expanded View Figures PDF

Table EV1

Table EV2

Source Data for Figure 6

## Data Availability

This study includes no data deposited in external repositories.
