# Supplementary material for: Conformational change of adenine nucleotide translocase‐1 mediates cisplatin resistance induced by EBV‐LMP1
Source: EMBO Mol Med. 2021 Nov 9;13(12):e14072. doi: 10.15252/emmm.202114072 (PMC8649884; doi:10.15252/emmm.202114072)
Supplement: Supplementary file 1 — Appendix [file EMMM-13-e14072-s006.pdf]

Appendix

Conformational change of adenine nucleotide translocase-1 mediates cisplatin resistance induced by EBV-LMP1

Table of content:
Appendix Table S1

Appendix Table S1. P-Value list.

Figure	p-value
Fig.1 A（CNE1 vs. CNE1-LMP1）	0.005
Fig.1 A（HK1 vs. HK1-LMP1）	0.039
Fig.1 B（CNE1 vs. CNE1-LMP1）	0.006
Fig.1 B（HK1 vs. HK1-LMP1）	0.004
Fig.1 C（CNE1 vs. CNE1-LMP1）	0.000
Fig.1 C（HK1 vs. HK1-LMP1）	0.001
Fig.1 D（CNE1 vs. CNE1-LMP1）	0.005
Fig.1 D（HK1 vs. HK1-LMP1）	0.002
Fig.3 B（Vector vs. BKA(10μM)）	0.009
Fig.3 B（Vector vs. CATR(20nM)）	0.000
Fig.3 D（Vector vs. LMP1(4ng)）	0.004
Fig.3 D（Vector vs. LMP1(8ng)）	0.003
Fig.4 B（Vehicle HK1 vs. HK1-LMP1）	0.000
Fig.4 B（Vehicle CNE1 vs. CNE1-LMP1）	0.001
Fig.4 D（HK1-CON vs. HK1-BKA）	0.000
Fig.4 D（HK1-CON vs. HK1-CATR）	0.000
Fig.4 D（HK1-CON vs. HK1-LMP1-CON）	0.049
Fig.4 D（HK1-LMP1-CON vs. HK1-LMP1-BKA）	0.003
Fig.4 D（HK1-LMP1-CON vs. HK1-LMP1-CATR）	0.001
Fig.4 D（CNE1-CON vs. CNE1-BKA）	0.000
Fig.4 D（CNE1-CON vs. CNE1-CATR）	0.000
Fig.4 D（CNE1-CON vs. CNE1-LMP1-CON）	0.042
Fig.4 D（CNE1-LMP1-CON vs. CNE1-LMP1-BKA）	0.005
Fig.4 D（CNE1-LMP1-CON vs. CNE1-LMP1-CATR）	0.004
Fig.5 B（CDDP vs. CON）	0.002
Fig.5 B（CDDP vs. BKA+CDDP）	0.048
Fig.5 B（CDDP vs. CATR+CDDP）	0.009
Fig.5 C（CDDP vs. CON）	0.000
Fig.5 C（CDDP vs. BKA+CDDP）	0.045
Fig.5 C（CDDP vs. CATR+CDDP）	0.021
Fig.5 F（CDDP vs. CON）	0.008
Fig.5 F（CDDP vs. CATR+CDDP）	0.005
Fig.5 F（CATR vs. CON）	0.046
Fig.5 F（CATR vs. CATR+CDDP）	0.003
Fig.5 G CNE1-LMP1（CON vs. CATR）	0.015
Fig.5 G CNE1-LMP1（CON vs. CDDP）	0.007
Fig.5 G CNE1-LMP1（CATR vs. CATR+CDDP）	0.005
Fig.5 G HK1-LMP1（CON vs. CATR）	0.044
Fig.5 G HK1-LMP1（CON vs. CDDP）	0.023
Fig.5 G HK1-LMP1（CATR vs. CATR+CDDP）	0.002
Fig.5 H CNE1-LMP1（CON vs. CATR）	0.008

Fig.5 H CNE1-LMP1（CON vs. CDDP）	0.005
Fig.5 H CNE1-LMP1（CATR vs. CATR+CDDP）	0.007
Fig.5 H HK1-LMP1（CON vs. CATR）	0.008
Fig.5 H HK1-LMP1（CON vs. CDDP）	0.000
Fig.5 H HK1-LMP1（CATR vs. CATR+CDDP）	0.005
Fig.6 C（Vehicle vs. CATR）	0.035
Fig.6 C（Vehicle vs. CDDP）	0.004
Fig.6 C（CDDP vs. CDDP+CATR）	0.042
Fig.6 D（Vehicle vs. CATR）	0.041
Fig.6 D（Vehicle vs. CDDP）	0.006
Fig.6 D（CDDP+CATR vs. CATR）	0.000
Fig.6 D（CDDP+CATR vs. CDDP）	0.004
Fig.6 E（CDDP vs. Vehicle）	0.001
Fig.6 E（CDDP vs. CDDP+CATR）	0.048
Fig.6 F（Vehicle vs. CATR）	0.045
Fig.6 F（Vehicle vs. CDDP）	0.007
Fig.6 F（CDDP+CATR vs. CATR）	0.000
Fig.6 F（CDDP+CATR vs. CDDP）	0.005
Fig.6 G（Vehicle vs. CATR）	0.011
Fig.6 G（Vehicle vs. CDDP）	0.008
Fig.6 G（CDDP+CATR vs. CATR）	0.002
Fig.6 G（CDDP+CATR vs. CDDP）	0.006
FigEV.1 B siRNA-CON （HK1 vs. HK1-LMP1)	0.025
FigEV.1 B siRNA-ANT1-2# （HK1 vs. HK1-LMP1)	0.074
FigEV.1 B siRNA-ANT1-3# （HK1 vs. HK1-LMP1)	0.171
FigEV.1 B siRNA-ANT2-1#（HK1 vs. HK1-LMP1)	0.016
FigEV.1 B siRNA-ANT2-2# （HK1 vs. HK1-LMP1)	0.017
FigEV.1 B siRNA-VDAC1-2# （HK1 vs. HK1-LMP1)	0.205
FigEV.1 B siRNA-VDAC1-3# （HK1 vs. HK1-LMP1)	0.401
FigEV.1 B siRNA-VDAC2-1# （HK1 vs. HK1-LMP1)	0.013
FigEV.1 B siRNA-VDAC2-3# （HK1 vs. HK1-LMP1)	0.017
FigEV.1 B siRNA-CypD-1#（HK1 vs. HK1-LMP1)	0.009
FigEV.1 B siRNA-CypD-3#（HK1 vs. HK1-LMP1)	0.001
FigEV.1 B siRNA-CON （CNE1 vs. CNE1-LMP1)	0.043
FigEV.1 B siRNA-ANT1-2# （CNE1 vs. CNE1-LMP1)	0.173
FigEV.1 B siRNA-ANT1-3# （CNE1 vs. CNE1-LMP1)	0.266
FigEV.1 B siRNA-ANT2-1#（CNE1 vs. CNE1-LMP1)	0.039
FigEV.1 B siRNA-ANT2-2# （CNE1 vs. CNE1-LMP1)	0.017
FigEV.1 B siRNA-VDAC1-2# （CNE1 vs. CNE1-LMP1)	0.165
FigEV.1 B siRNA-VDAC1-3# （CNE1 vs. CNE1-LMP1)	0.023
FigEV.1 B siRNA-VDAC2-1# （CNE1 vs. CNE1-LMP1)	0.013
FigEV.1 B siRNA-VDAC2-3# （CNE1 vs. CNE1-LMP1)	0.048
FigEV.1 B siRNA-CypD-1#（CNE1 vs. CNE1-LMP1)	0.011
FigEV.1 B siRNA-CypD-3#（CNE1 vs. CNE1-LMP1)	0.006
FigEV.2 D TMRM-CON (HK1 vs. HK1-LMP1)	0.004
FigEV.2 D TMRM-CATR (HK1 vs. HK1-LMP1)	0.021
FigEV.2 D TMRM-CON (CNE1 vs. CNE1-LMP1)	0.002
FigEV.2 D TMRM-CATR (CNE1 vs. CNE1-LMP1)	0.282
FigEV.2 D Cell viability-CON (HK1 vs. HK1-LMP1)	0.007
FigEV.2 D Cell viability-CATR (HK1 vs. HK1-LMP1)	0.821
FigEV.2 D Cell viability-CON (CNE1 vs. CNE1-LMP1)	0.001

**FigEV.2 D Cell viability-CATR (CNE1 vs. CNE1-LMP1)**	**0.782**
**FigEV.2 E TMRM-CON (HK1 vs. HK1-LMP1)**	**0.004**
**FigEV.2 E TMRM-BKA (HK1 vs. HK1-LMP1)**	**0.549**
**FigEV.2 E TMRM-CON (CNE1 vs. CNE1-LMP1)**	**0.008**
**FigEV.2 E TMRM-BKA (CNE1 vs. CNE1-LMP1)**	**0.620**
**FigEV.2 E Cell viability-CON (HK1 vs. HK1-LMP1)**	**0.041**
**FigEV.2 E Cell viability-BKA (HK1 vs. HK1-LMP1)**	**0.611**
**FigEV.2 E Cell viability-CON (CNE1 vs. CNE1-LMP1)**	**0.046**
**FigEV.2 E Cell viability-BKA (CNE1 vs. CNE1-LMP1)**	**0.588**
**FigEV.2 F HK1-LMP1 (CATR(-) vs. CATR(+))**	**0.000**
**FigEV.2 F HK1-LMP1 (BKA(-) vs. BKA(+))**	**0.355**
**FigEV.2 F CNE1-LMP1 (CATR(-) vs. CATR(+))**	**0.001**
**FigEV.2 F CNE1-LMP1 (BKA(-) vs. BKA(+))**	**0.401**
**FigEV.3 C (CDDP vs. CON)**	**0.005**
**FigEV.3 C (CDDP vs. BKA+CDDP)**	**0.047**
**FigEV.3 C (CDDP vs. CATR+CDDP)**	**0.008**
**FigEV.3 D (CDDP vs. CON)**	**0.000**
**FigEV.3 D (CDDP vs. BKA+CDDP)**	**0.021**
**FigEV.3 D (CDDP vs. CATR+CDDP)**	**0.009**
**FigEV.3 E (CDDP vs. CON)**	**0.000**
**FigEV.3 E (CDDP vs. BKA+CDDP)**	**0.043**
**FigEV.3 E (CDDP vs. CATR+CDDP)**	**0.007**
**FigEV.3 H (CDDP vs. CON)**	**0.048**
**FigEV.3 H (CDDP vs. CATR+CDDP)**	**0.007**
**FigEV.3 H (CATR vs. CON)**	**0.009**
**FigEV.3 H (CATR vs. CATR+CDDP)**	**0.002**
