# Supplementary material for: Conformational change of adenine nucleotide translocase‐1 mediates cisplatin resistance induced by EBV‐LMP1
Source: EMBO Mol Med. 2021 Nov 9;13(12):e14072. doi: 10.15252/emmm.202114072 (PMC8649884; doi:10.15252/emmm.202114072)
Supplement: Supplementary file 2 — Expanded View Figures PDF [file EMMM-13-e14072-s003.pdf]

Expanded View Figures

Figure EV1. EBV-LMP1 interacting with ANT1 localizes to mitochondria.

A Knockdown effect of mPTP complex components.
B Effect of knockdown of each mPTP component on the membrane potential of EBV-LMP1-negative or EBV-LMP1-positive nasopharyngeal carcinoma cells. Data are presented as means ± SEM (paired *t*-test, $n = 5$, biological replicates per group, $*P < 0.05$, $**P < 0.01$).
C, D The specificity of ANT1 antibody was verified in this experiment using: knockdown or overexpression of ANT1 in HK1 cells, respectively (C); expression of HK1-derived ANT1 in prokaryotes and validation after purification (D).
E LMP1 is present in the cytoplasm and mitochondria. EBV-LMP1-positive nasopharyngeal carcinoma cells were isolated from cytoplasm and mitochondria and western blot assayed for LMP1 expression.
F Co-IP detection of the interaction of EBV-LMP1 and ANT1 in CNE1-LMP1 cells.
G Laser confocal analysis of CNE1-LMP1 cells revealed the presence of co-localization of LMP1 with ANT1 (scale bar, 5 μm). The quantified graph on the lower right shows the percentage of yellow fluorescence (merge) to red fluorescence (ANT1) in CNE1-LMP1 cells. Data are presented as means ± SEM ($n = 6$, biological replicates per group).
H PLA detects the presence of direct binding of LMP1 to ANT1, but not VDAC1, in CNE1-LMP1 cells (scale bar, 10 μm).

 © 2021 The Authors.

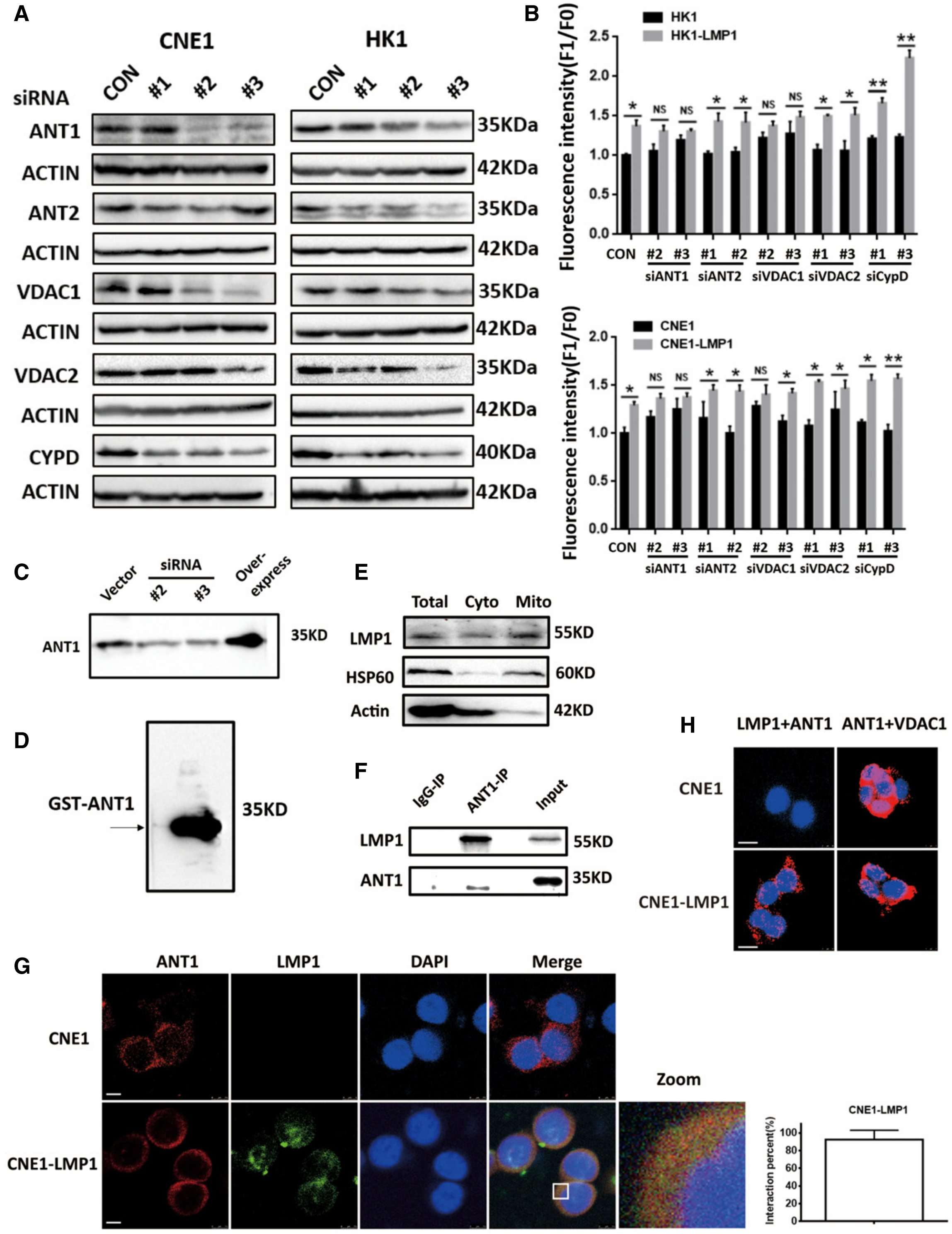
A
CNE1
HK1
siRNA
CON
#1
#2
#3
ANT1
ACTIN
ANT2
VDAC1
VDAC2
CYPD
35KDa
42KDa
40KDa
B
HK1
HK1-LMP1
Fluorescence intensity(F1/F0)
siANT1
siANT2
siVDAC1
siVDAC2
siCypD
CNE1
CNE1-LMP1
NS
C
Vector
siRNA
Over-express
35KD
D
GST-ANT1
E
Total
Cyto
Mito
LMP1
HSP60
Actin
55KD
60KD
42KD
F
IgG-IP
ANT1-IP
Input
H
LMP1+ANT1
ANT1+VDAC1
G
DAPI
Merge
Zoom
Interaction percent(%)

Figure EV1.

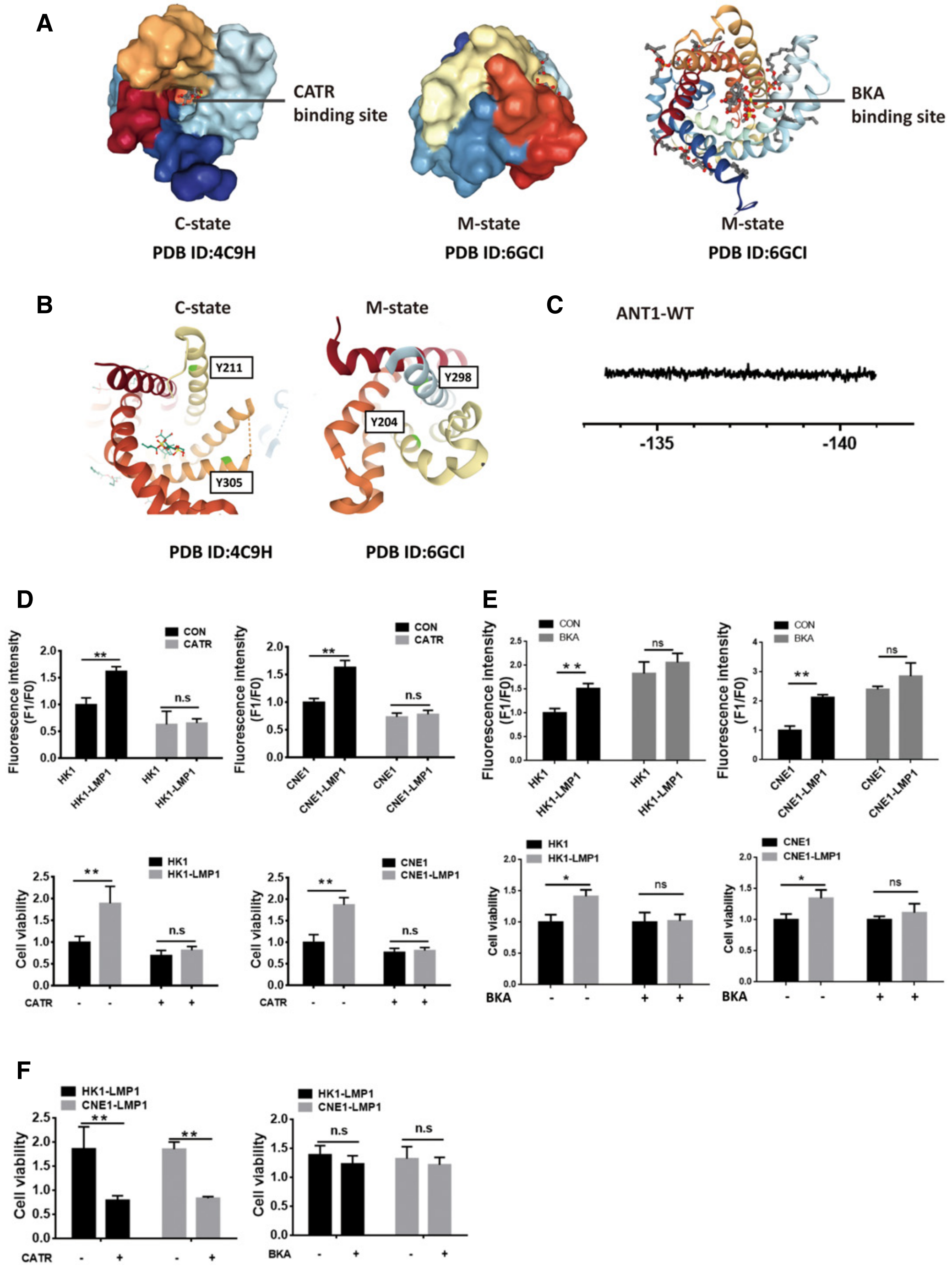

Figure EV2. Effect of BKA and CATR inhibitors on nasopharyngeal carcinoma cell function.

A Binding sites of BKA and CATR inhibitors to the ADP/ATP transporter.

B Spatial position of Y195 and Y290 in two different conformations of ANT1. Y195 (yeast: Y211; *Saccharomyces cerevisiae*: Y204) and Y290 (yeast: Y305; *Saccharomyces cerevisiae*: Y298).

C 19F-NMR assay of ANT1-WT as a reference.

D Effects of CATR on nasopharyngeal carcinoma cell mitochondrial membrane potential and cell viability. "–" represents vehicle treatment only. Data are presented as means ± SEM (paired *t*-test, $n = 6$, biological replicates per group, $**P < 0.01$).

E Effects of BKA on nasopharyngeal carcinoma cell mitochondrial membrane potential and viability. "–" represents vehicle treatment only. Data are presented as means ± SEM (paired *t*-test, $n = 6$, biological replicates per group, $*P < 0.05$, $**P < 0.01$).

F Cell viability assay in LMP1-positive NPC cells in CATR/BKA untreated group vs. treated group. "–" represents vehicle treatment only. Data are presented as means ± SEM (paired *t*-test, $n = 6$, biological replicates per group, $**P < 0.01$).

 © 2021 The Authors.

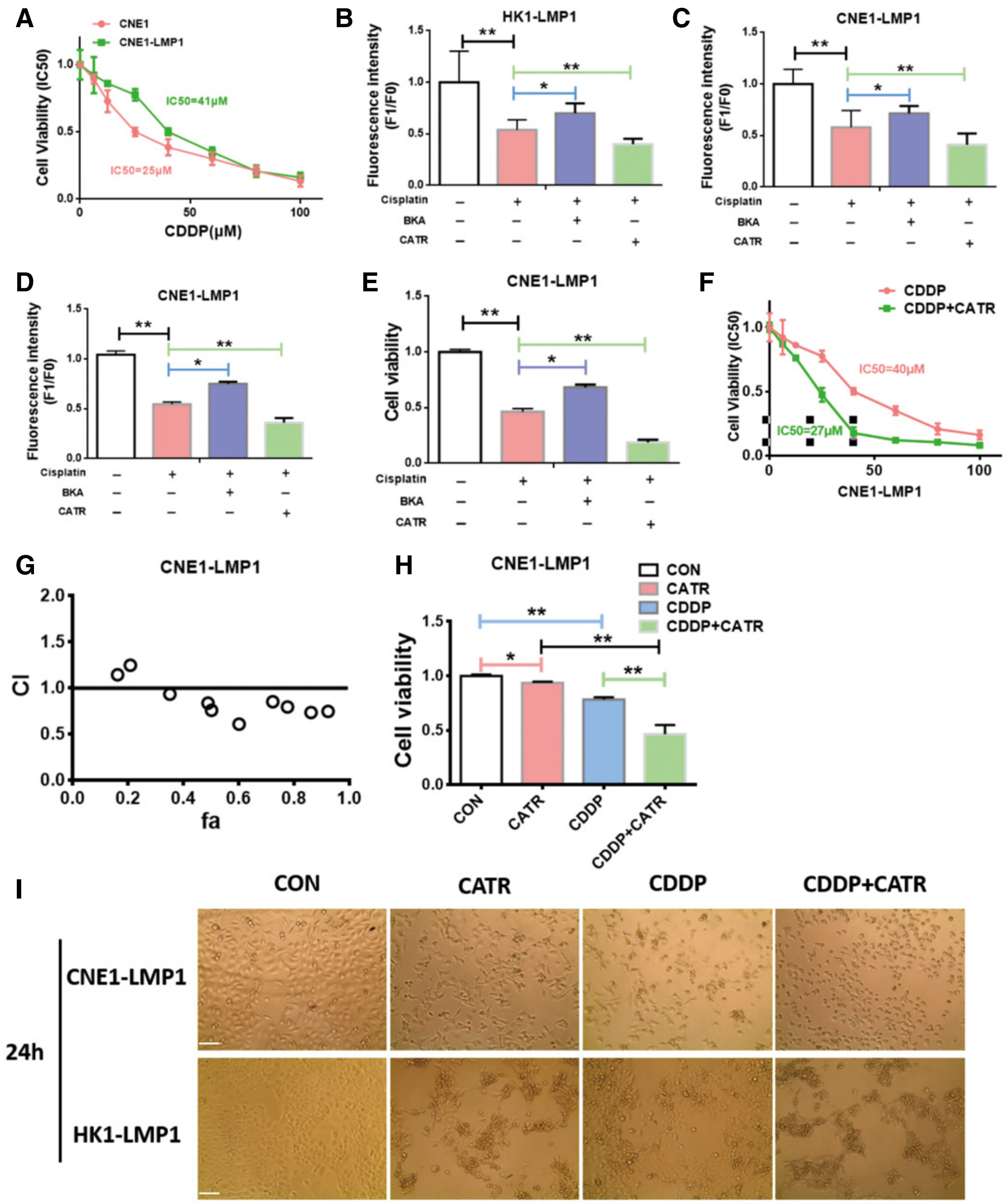

Figure EV3. CATR enhances the sensitivity of NPC cells to cisplatin.

A CNE1 and CNE1-LMP1 cells were treated for 24 h with increasing concentrations of cisplatin. Cell viability was determined by MTS assay. Data are presented as means ± SEM ($n = 6$, biological replicates per group).

B, C LMP1-positive NPC cells were treated with cisplatin for 24 h and BKA (1 μM)/CATR was added for immediate detection of mitochondrial membrane potential. Data are presented as means ± SEM (paired t-test, $n = 6$, biological replicates per group, $^{*}P < 0.05$, $^{**}P < 0.01$).

D, E Changes in mitochondrial membrane potential and viability of and CNE1-LMP1 cells treated for 24 h with cisplatin (20 μM) alone or cisplatin (20 μM) combined with BKA (1 μM)/CATR (1 μM). Mitochondrial membrane potential of cells was analyzed by using flow cytometry (B), and viability was measured by the CCK-8 assay (C). Data are presented as means ± SEM (paired t-test, $n = 6$, biological replicates per group, $^{*}P < 0.05$, $^{**}P < 0.01$).

F The IC_{50} value of the combination of CATR and cisplatin in CNE1-LMP1 was changed. Data are presented as means ± SEM ($n = 6$, biological replicates per group).

G The combination index of cisplatin and CATR in CNE1-LMP1 cells (note: CI < 1 indicates synergism; CI = 1 indicates an additive effect; and CI > 1 indicates antagonism).

H CCK-8 analysis of viability in CNE1-LMP1 cells treated for 24 h with CATR (1 μM) alone, cisplatin (20 μM) alone, or cisplatin (20 μM) combined with CATR (1 μM). Data are presented as means ± SEM (paired t-test, $n = 6$, biological replicates per group, $^{*}P < 0.05$, $^{**}P < 0.01$).

I Morphology of NPC cells treated for 24 h with vehicle, cisplatin, CATR, or a combination of cisplatin and CATR (scale bar, 100 μm).

© 2021 The Authors.

 © 2021 The Authors.
