# Supplementary material for: Conformational change of adenine nucleotide translocase‐1 mediates cisplatin resistance induced by EBV‐LMP1
Source: EMBO Mol Med. 2021 Nov 9;13(12):e14072. doi: 10.15252/emmm.202114072 (PMC8649884; doi:10.15252/emmm.202114072)
Supplement: Supplementary file 6 [file EMMM-13-e14072-s002.pdf]

EMBO Molecular Medicine

Conformational change of adenine nucleotide translocase-1 mediates cisplatin resistance induced by EBV-LMP1

Lin Zhao, Xiangying Deng, Yueshuo Li, Jianmin Hu, longlong xie, Feng Shi, Min Tang, Ann Bode, Xin Zhang, Weihua Liao, and Ya Cao
DOI: 10.15252/emmm.202114072

Corresponding author: Ya Cao (ycao98@vip.sina.com.cn)

Review Timeline:

Submission Date:	3rd Feb 21
Editorial Decision:	11th Mar 21
Editor Correspondence:	18th Mar 21
Revision Received:	11th Aug 21
Editorial Decision:	9th Sep 21
Revision Received:	20th Sep 21
Accepted:	29th Sep 21

Editor: Lise Roth

Transaction Report:

(Note: With the exception of the correction of typographical or spelling errors that could be a source of ambiguity, letters and reports are not edited. Depending on transfer agreements, referee reports obtained elsewhere may or may not be included in this compilation. Referee reports are anonymous unless the Referee chooses to sign their reports.)

11th Mar 2021

Dear Prof. Cao,

Thank you for the submission of your manuscript to EMBO Molecular Medicine, and please accept my apologies for the delay in getting back to you, which is due to the fact that we were waiting for the report from the third referee. As we have not received this report yet and given that both referees #1 and #2 are overall positive, we prefer to make a decision now in order to avoid further delay in the process. Should referee #3 provide a report shortly, we will send it to you, with the understanding that we would not ask you for extensive experiments in addition to the ones required in the enclosed reports.

As you will see from the reports below, the referees acknowledge the interest of the study and are overall supporting publication of your work pending appropriate revisions.
Addressing the reviewers' concerns in full will be necessary for further considering the manuscript in our journal. Acceptance of the manuscript will entail a second round of review. EMBO Molecular Medicine encourages a single round of revision only and therefore, acceptance or rejection of the manuscript will depend on the completeness of your responses included in the next, final version of the manuscript. For this reason, and to save you from any frustrations in the end, I would strongly advise against returning an incomplete revision.

When submitting your revised manuscript, please carefully review the instructions that follow below. Failure to include requested items will delay the evaluation of your revision:

1) A .docx formatted version of the manuscript text (including legends for main figures, EV figures and tables). Please make sure that the changes are highlighted to be clearly visible.

2) Individual production quality figure files as .eps, .tif, .jpg (one file per figure).

3) A .docx formatted letter INCLUDING the reviewers' reports and your detailed point-by-point responses to their comments. As part of the EMBO Press transparent editorial process, the point-by-point response is part of the Review Process File (RPF), which will be published alongside your paper.

4) A complete author checklist, which you can download from our author guidelines (https://www.embopress.org/page/journal/17574684/authorguide#submissionofrevisions). Please insert information in the checklist that is also reflected in the manuscript. The completed author checklist will also be part of the RPF.

5) Please note that all corresponding authors are required to supply an ORCID ID for their name upon submission of a revised manuscript.

6) Before submitting your revision, primary datasets produced in this study need to be deposited in an appropriate public database (see https://www.embopress.org/page/journal/17574684/authorguide#dataavailability).
Please remember to provide a reviewer password if the datasets are not yet public. The accession numbers and database should be listed in a formal "Data Availability " section (placed after Materials & Method). Please note that the Data Availability Section is restricted to new primary data that are part of this study.

All links should resolve to a page where the data can be accessed.

7) We would also encourage you to include the source data for figure panels that show essential data. Numerical data should be provided as individual .xls or .csv files (including a tab describing the data). For blots or microscopy, uncropped images should be submitted (using a zip archive if multiple images need to be supplied for one panel). Additional information on source data and instruction on how to label the files are available at
.

8) Our journal encourages inclusion of *data citations in the reference list* to directly cite datasets that were re-used and obtained from public databases. Data citations in the article text are distinct from normal bibliographical citations and should directly link to the database records from which the data can be accessed. In the main text, data citations are formatted as follows: "Data ref: Smith et al, 2001" or "Data ref: NCBI Sequence Read Archive PRJNA342805, 2017". In the Reference list, data citations must be labeled with "[DATASET]". A data reference must provide the database name, accession number/identifiers and a resolvable link to the landing page from which the data can be accessed at the end of the reference. Further instructions are available at .

9) We replaced Supplementary Information with Expanded View (EV) Figures and Tables that are collapsible/expandable online. A maximum of 5 EV Figures can be typeset. EV Figures should be cited as 'Figure EV1, Figure EV2" etc... in the text and their respective legends should be included in the main text after the legends of regular figures.

- For the figures that you do NOT wish to display as Expanded View figures, they should be bundled together with their legends in a single PDF file called *Appendix*, which should start with a short Table of Content. Appendix figures should be referred to in the main text as: "Appendix Figure S1, Appendix Figure S2" etc.

- Additional Tables/Datasets should be labeled and referred to as Table EV1, Dataset EV1, etc. Legends have to be provided in a separate tab in case of .xls files. Alternatively, the legend can be supplied as a separate text file (README) and zipped together with the Table/Dataset file.
See detailed instructions here:
.

10) The paper explained: EMBO Molecular Medicine articles are accompanied by a summary of the articles to emphasize the major findings in the paper and their medical implications for the non-specialist reader. Please provide a draft summary of your article highlighting
- the medical issue you are addressing,
- the results obtained and
- their clinical impact.

This may be edited to ensure that readers understand the significance and context of the research. Please refer to any of our published articles for an example.

11) For more information: There is space at the end of each article to list relevant web links for further consultation by our readers. Could you identify some relevant ones and provide such information as well? Some examples are patient associations, relevant databases, OMIM/proteins/genes links, author's websites, etc...

12) Every published paper now includes a 'Synopsis' to further enhance discoverability. Synopses are displayed on the journal webpage and are freely accessible to all readers. They include a short stand first (maximum of 300 characters, including space) as well as 2-5 one-sentences bullet points that summarizes the paper. Please write the bullet points to summarize the key NEW findings. They should be designed to be complementary to the abstract - i.e. not repeat the same text. We encourage inclusion of key acronyms and quantitative information (maximum of 30 words / bullet point). Please use the passive voice. Please attach these in a separate file or send them by email, we will incorporate them accordingly.

Please also suggest a striking image or visual abstract to illustrate your article. If you do please provide a png file 550 px-wide x 400-px high.

13) As part of the EMBO Publications transparent editorial process initiative (see our Editorial at http://embomolmed.embopress.org/content/2/9/329), EMBO Molecular Medicine will publish online a Review Process File (RPF) to accompany accepted manuscripts.
In the event of acceptance, this file will be published in conjunction with your paper and will include the anonymous referee reports, your point-by-point response and all pertinent correspondence relating to the manuscript. Let us know whether you agree with the publication of the RPF and as here, if you want to remove or not any figures from it prior to publication.
Please note that the Authors checklist will be published at the end of the RPF.

EMBO Molecular Medicine has a "scooping protection" policy, whereby similar findings that are published by others during review or revision are not a criterion for rejection. Should you decide to submit a revised version, I do ask that you get in touch after three months if you have not completed it, to update us on the status.

I look forward to receiving your revised manuscript.

Yours sincerely,

Lise Roth

Lise Roth, PhD
Editor
EMBO Molecular Medicine

***** Reviewer's comments *****

Referee #1 (Remarks for Author):

The paper by Zhao et al. deals with the role of adenine nucleotide translocase-1 (ANT1), a death-stimulatory isoform of ANT, in the response of nasopharygeal carcinomal cell lines expressing the Epstein Barr virus (EBV)-encoded latent membrane protein 1 (LMP1) to cisplatin-based chemotherapy in vitro. The authors show that LMP1 physically interacts with ANT1, forcing into a confirmation (the m-state) that is incompatible with the opening of the mitochondrial permeability transition pore (mPTP), hence rendering the cell resistant to cisplatin-induced cell death. However, carboxyatractyloside (CATR), which induces a pro-mPTP conformation of ANT1 (the c-state) can reestablish the response of IMP1-expressing cells to cisplatin. This is shown both in vitro and in vivo, in mice bearing xengrafted human cancer cell lines. Altogether, this a convincing story supported by an orthogonal approach, careful experimental design and compelling data. The authors should consider to improve their paper in the following points:

Results:

• Explain the two sets of NPC cells (CNE1/CNE1-LMP1 and HK1/HK1-LMP1) the first time that you mention them.

• Fig. 1c lacks replicates and statistical comparisons

• Fig. 2A,B. Are these results obtained from the same membrane?

• All panels of Fig. 2. Quantifications and statistical comparisons are missing.

• Fig. 3A,B. Quantifations and statistical comparisons are missing.

• Fig. 4C: Statistical comparisons are missing.

• Fig. 6D, F, G: Statistical comparisons are missing between the CATR and the CATR+CDDP groups (the same applies for the corresponding supplemental figure)

• Fig; 6: Have the in vivo experiments been performed several times?

• It should be noted that the results in vivo (on tumor growth) appear additve, not synergistic. This should be mentioned as a caveat in the discussion.

Minor:

Avoid unexplained abbreviations in the abstract (CATR).

Define what JC-1 stands for

"the mPTP allows free passage of substances less than or equal to 1.5 KD". Replace "substances" by "solutes"

Define what means "significantly" in the Results (p value and statistical test)

It is not common language to say that mice were inoculated into the "armpit".

• Fig. 7: Please indicate the two different ANT1 conformations (m-state versus c-state)

Referee #2 (Remarks for Author):

It is pressing to elucidate the mechanism of drug resistance for new cancer treatment approaches and strategies. In this regard, the authors of this study identified a novel role for EBV-LMP1-induced ANT1 conformation changes in cisplatin resistance of nasopharyngeal carcinoma. They found that EBV-LMP1 localizes to the inner mitochondrial membrane and inhibits the opening of mPTP by binding to ANT1 fixed to m-state, thereby favoring tumor cell survival and drug resistance. On the whole, the major

findings of this research are novel and interesting. But the manuscript may need some revisions before publication.
1. The titles of the figures are kind of different from the subheads of the manuscript. The authors should check them carefully to make sure of the consistency between the conclusions and the data provided in the figures, especially the titles of Figure 4 and Figure 6.
2. In Figure 2, the authors proved that ANT1 binds to LMP1 and identified the specific binding regions, but they didn't provide evidence to show that the binding of these two proteins is necessary for the conformation change of ANT1.The authors may need further experiments or an interpretation in this regard.
3. In Figure 3A, the results indicate that BKA, an inhibitor keeping ANT1 in m-state, inhibits the binding of ANT1 and VDAC1, while CATR, an inhibitor stabilizing ANT1 in c-state, promotes the binding. But the authors drew a totally opposite conclusion in the manuscript (line 211-214), which should be corrected.
4. In Figure 3F, this reviewer would expect that the cell viability in BKA treatment groups is comparable with that in LMP1-overexprssed cells without BKA treatment because ANT1 is at m-state in these three groups, but it was not the case as shown in the results. Besides, it's better to use CATR to attenuate ANT1 at c-state to prove LMP1 modulate mitochondrial membrane potential and cell viability by keeping ANT1 at m-state.
5. The authors should check through the manuscript carefully to correct typo errors and other mistakes. Following are some examples:1) The figure legend of Fig 2E indicated that the experiment was carried out in 293T, but the results shown in the figure were not in 293T; 2) The antibodies used for IP in Fig 2F and Fig 2G are reversed, making the results confusing; 3) Label "G" was missing and label "F" was in a wrong place in Figure 2; 4) "7-ADD" should be corrected as "7-AAD" in Fig 5G and the manuscript; 5) The results and methods of Figure 6 showed that the xenograft experiment used up to 5×107 cells, which was far beyond common sense. Please check through the texts to correct these mistakes and make sure that the figure legends and methods contain enough information to allow interpretation and replication of the results.

Dear Prof. Cao,

We have now received the report from reviewer #3, that you will find attached to this email. As mentioned in my decision letter, we would like you to address the issues raised by this referee, but do not ask for further reaching experiments. Specifically, we would like you to address the points 1, 3, 6, 7, 8, and 9 (CDDP) in writing. Please also improve the quality of the figures and their description throughout the manuscript, as mentioned in point 4. Regarding point 2, we would be happy for you to include additional experiments, or at least discuss the limitations of the method as mentioned by the referee. Please address experimentally points 5 and 9 (IP).

Do not hesitate to contact me should you have any question.

Looking forward to receiving your revised manuscript,

Sincerely,

Lise Roth

Lin Zhao and colleagues aimed to identify mechanism by which a latent membrane protein 1 (LMP1), encoded by Epstein-Barr virus (EBV), induces alteration of ANT1 conformation in cisplatin (CDDP)-resistant nasopharyngeal carcinoma cells (NPC).
The authors described that EBV-LMP1 inhibits the mPTP opening by binding to ANT1, leading to the increased tumour cell survival and drug resistance. ANT1 inhibitor CATR and especially its combination with CDDP improved a chemosensitivity of EBV-LMP1-positive cells.
In my opinion several obtained results not necessarily support the made conclusion, the interpretation of several experiments is not convincing and important controls are lacking. The manuscript itself reminds me of the first draft.
Major comments
1. The introduction describes the current research field very narrowly and subjectively. The fact that the ANT1 is a part of mPTP is highly controversial. Zhao et al. didn´t mention the existing hot debates and other hypotheses, excluding ANT1.
ANT1 was shown to be not only ATP/ADP transporter, but obviously also participates in the proton transport in the presence of fatty acids. As recently shown, the proton transport is also affected by CATR and BA.
2. Several very important conclusions are based on the measurements of mitochondrial membrane potential (MMP) using fluorescent dyes TMRM and JC1.
However, only measurements which investigate the immediate MMP changes after direct addition of substances potential are easy to interpret. The comparison of two different cell cultures (e.g. wt and KO or cells before and after 24 h incubation (Fig. 5 B/C)) is very challenging because of the different dye/cell concentration, change in mitochondrial surface/volume ratio and loading time.
3. It is not clear how the authors calculated the fluorescent intensity ratio F/F0 (e.g. Fig. 1, A)? How they have taken in account a different amount of cells in each snapshot? Please explain, how you perform statistics in the experiment shown in Fig. 1, D? My concern is that the fluorescence will depend on the cell amount.
4. The scientific quality of the figures and their descriptions is below the usual requirements for a bachelor thesis. Sometimes I get the impression that the authors do not know exactly what they are measuring. This applies to all Figures. I will give only some examples below.
Fig. 1, B: y-axis is entitled scientifically incorrect!
Fig. 1, C: x-axis is entitled scientifically incorrect!
Line 141 I don´t see how the conclusion "suggesting that EBV-LMP1 ..... increases the mitochondrial membrane potential" can be made from Fig. 1C.
The experiment shown in Fig. 1D should be explain better. The use of ionomycin is controversial (PMID: 28655872), the authors should take better A23187, to induce a decrease in calcein fluorescence.
Suppl. Fig. 1 Actin is not appropriately shown in Western Blots and cannot be used as a control.
Please explain what we see in the Western Blot (?) at 35 kD. There is no appropriate description of the Fig. 3D.
Suppl. Fig. 2C. The line description: "19F-NMR assay of ANT1-WT" doesn´t help to understand what was measured.
5. In several experiments the antibodies (AB) against ANT1 and ANT2 are used. Due to relatively high homology between mitochondrial transporters AB against ANT1 are known to be highly unspecific and cannot be used in immunohistochemical staining. Authors should perform (i) full size WB for ANT1 expression in HK1 cell to validate the use of the antibody for staining and (ii) to show the specificity of this AB using ANT1 KO cells/tissues as

negative control. Also appropriate positive control (e.g. recombinant ANT1) is required.
6. Which absolute values for ATP/ADP exchange were measured in the experiments shown in Fig. 4b? How this exchange was measured?
7. Please explain how the Seahorse experiment justifies the conclusion "the process is closely related to ANT1 conformation-based functional restrictions." (Fig. 4, C).
8. What is the hypothesis for the usage of two sets of NPC cells?
9. A representative IP for LPM1 (Fig. 3 B) is not convincing. Authors should show at least two other experiments.
9. CDDP (cis-diamminedichloroplatinum? this abbreviation is not explained in the text) and cisplatin are obviously the same substances. However, authors describe their experiment like this (Line 291): "... cells were divided into a control group, CDDP-treatment only group, cisplatin-treatment only group, and a combination of CDDP and cisplatin-treated group, respectively." This description should be corrected.

***** Reviewer's comments *****

Reviewer #1:

The paper by Zhao et al. deals with the role of adenine nucleotide translocase-1 (ANT1), a death-stimulatory isoform of ANT, in the response of nasopharygeal carcinomal cell lines expressing the Epstein Barr virus (EBV)-encoded latent membrane protein 1 (LMP1) to cisplatin-based chemotherapy in vitro. The authors show that LMP1 physically interacts with ANT1, forcing into a confirmation (the m-state) that is incompatible with the opening of the mitochondrial permeability transition pore (mPTP), hence rendering the cell resistant to cisplatin-induced cell death. However, carboxyatractyloside (CATR), which induces a pro-mPTP conformation of ANT1 (the c-state) can reestablish the response of lMP1-expressing cells to cisplatin. This is shown both in vitro and in vivo, in mice bearing xengrafted human cancer cell lines. Altogether, this a convincing story supported by an orthogonal approach, careful experimental design and compelling data. The authors should consider to improve their paper in the following points:

• Explain the two sets of NPC cells (CNE1/CNE1-LMP1 and HK1/HK1-LMP1) the first time that you mention them.

Author Response: The NPC cell line CNE1 was purchased from the Cell Resource Center of Central South University. HK1 were generously provided by Professor Sai Wah Tsao from the University of Hong Kong. CNE1-LMP1 and HK1-LMP1 cells were established by our laboratory and cultured in RPMI-1640 medium with 10% fetal bovine serum (line 430-434).

• Fig. 1C lacks replicates and statistical comparisons

Author Response: The statistical comparison information has been supplemented in the figure with the following results.

Fig. 1C:

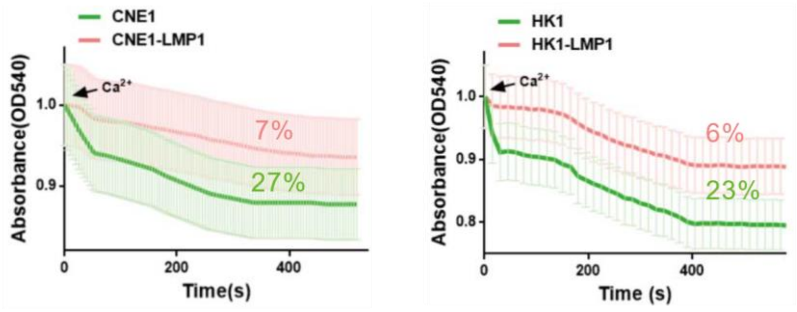

• Fig. 2A,B. Are these results obtained from the same membrane?

Author Response: Fig. 2A is the western blot result of mitochondria and mitochondrial membrane, and Fig. 2B is the result of NPC cell confocal. The two western results of Fig.2A are not derived from the same membrane: Fig.2A(left) is isolated mitochondria from LMP1-positive cells; Fig.2A (right) is mitochondrial and membrane components from LMP1-positive cells.

Fig. 2A:

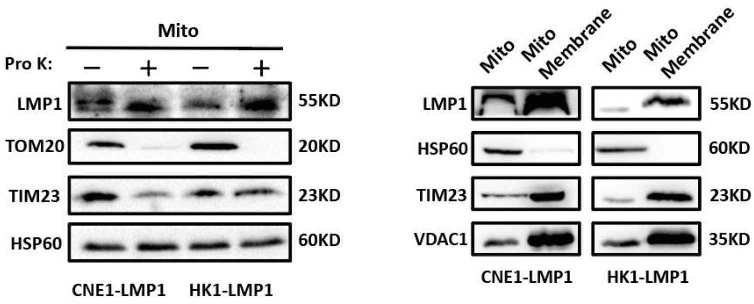

Fig. 2B:

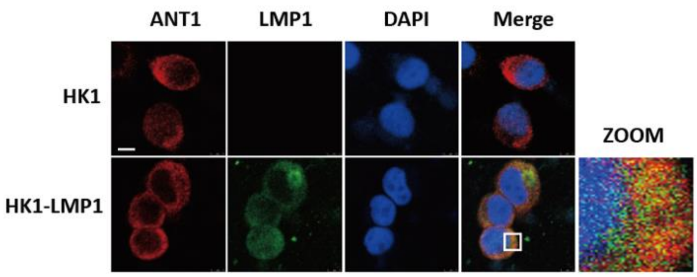

• All panels of Fig. 2. Quantifications and statistical comparisons are missing.

Author Response: Fig. 2 No statistical analysis could be performed.

• Fig. 3A, B. Quantifations and statistical comparisons are missing.

Author Response: Quantitative and statistical comparisons were added in the revised manuscript: Figure 3A in the original figure is Figure 3B in the revised manuscript, and Figure 3B in the original figure is Figure 3D in the revised manuscript.

Fig. 3A:

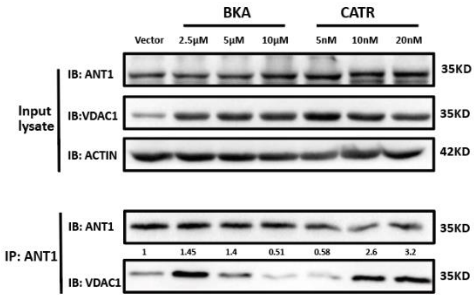

Fig. 3B:

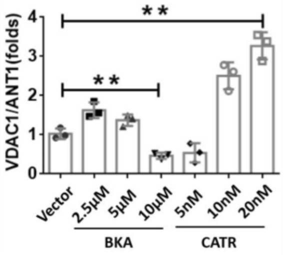

Fig. 3C:

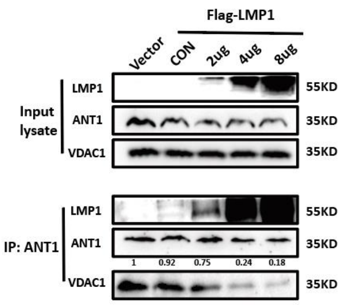

Fig. 3D:

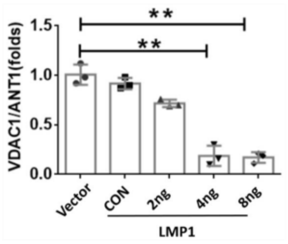

• Fig. 4C: Statistical comparisons are missing.

Author Response: The oxidative phosphorylation-related indicators based on OCR data have been statistically analyzed in the revised manuscript, and the results are shown in the following figures:

Fig. 4D:

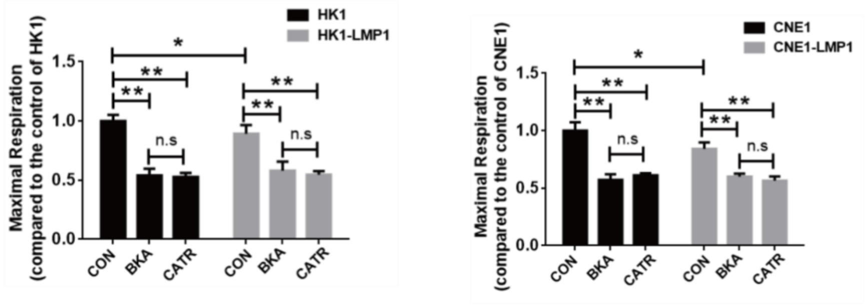

• Fig. 6D, F, G: Statistical comparisons are missing between the CATR and the CATR+CDDP groups (the same applies for the corresponding supplemental figure)

Author Response: Statistical comparisons between the CATR and CATR+CDDP groups in Figures 6D, F, G, and Supplementary Figure 3H have been refined and the results are shown in the following figures.

Figures 6D:

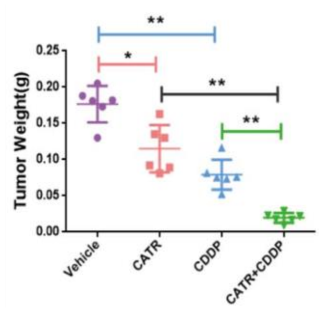

Figures 6F:

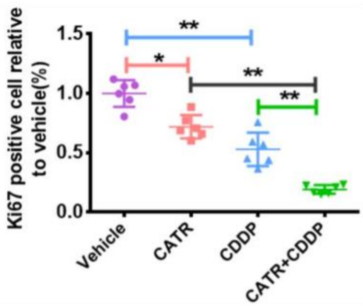

Figures 6G:

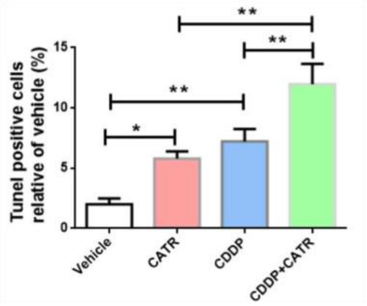

Supplementary Figure 3H:

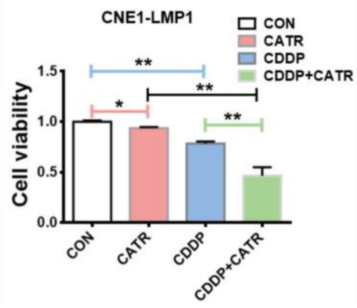

• Fig; 6: Have the in vivo experiments been performed several times?

Author Response: The mouse transplantation tumor experiments were divided into 4 groups of 6 mice each for parallel experiments.

• It should be noted that the results in vivo (on tumor growth) appear additve, not synergistic. This should be mentioned as a caveat in the discussion.

Author Response: We examined the CI values of HK1-LMP1 and CNE1-LMP1 according to the method shown in the Chou-Talalay equation (Chou & Talalay, 1983, Tsai, Liu et al., 2018) (line565), which seems to be applicable only to in vitro experiments. We have not yet found a method applicable to the calculation of CI values in vivo, so it is not appropriate to discuss further on the basis of the existing experiments at this time.

• Fig. 7: Please indicate the two different ANT1 conformations (m-state versus c-state)

Author Response: ANT has two different conformations: when ANT1 is facing the

mitochondrial matrix, it transports ATP to release ADP, which is in the m-state, and when ANT1 is facing the cytoplasmic side, it transports ADP to release ATP, which is in the c-state; ANT achieves mitochondrial energy conversion through a cyclic state transition, the inhibitors CATR and bongkrekic acid (BKA) fix ANT1 in the c-state and m-state, respectively (line 74-79).

（2）Minor:

Avoid unexplained abbreviations in the abstract (CATR).

Author Response: The ANT1 conformational inhibitor carboxyatractyloside (CATR) in combination with cisplatin improved the chemosensitivity of EBV-LMP1-positive cells (line 46).

Define what JC-1 stands for "the mPTP allows free passage of substances less than or equal to 1.5 KD". Replace "substances" by "solutes"

Author Response: Under normal physiological conditions, the mPTP allows free passage of solutes armpit less than or equal to 1.5 KD (line 133).

Define what means "significantly" in the Results (p value and statistical test)

Author Response: The statistical significance of the data was analyzed by using a standard Student’s t-test. A p-value of < 0.05 was deemed statistically significant and $p< 0.01$ was considered statistically significant (line 644).

It is not common language to say that mice were inoculated into the "armpit".

Author Response: The revised manuscript has been modified to inoculate subcutaneously into mice (line 630-631).

Reviewer #2:

It is pressing to elucidate the mechanism of drug resistance for new cancer treatment approaches and strategies. In this regard, the authors of this study identified a novel role for EBV-LMP1-induced ANT1 conformation changes in cisplatin resistance of nasopharyngeal carcinoma. They found that EBV-LMP1 localizes to the inner mitochondrial membrane and inhibits the opening of mPTP by binding to ANT1 fixed to m-state, thereby favoring tumor cell survival and drug resistance. On the whole, the major findings of this research are novel and interesting. But the manuscript may need some revisions before publication.

1. The titles of the figures are kind of different from the subheads of the manuscript. The authors should check them carefully to make sure of the consistency between the conclusions and the data provided in the figures, especially the titles of Figure 4 and Figure 6.

Author Response: The titles of the figures (Figure 4 and Figure 6) and the subtitle of the manuscript have been revised to be consistent in the revised manuscript (line 268/334).

2. In Figure 2, the authors proved that ANT1 binds to LMP1 and identified the specific binding regions, but they didn't provide evidence to show that the binding of these two proteins is necessary for the conformation change of ANT1.The authors may need further experiments or an interpretation in this regard.

Author Response: We constructed a prokaryotic expression vector for LMP1 (LMP1-ΔT) with deletion of the transmembrane domain (LMP1 binds to ANT1 through this domain), and after its prokaryotic expression in the strain and purification, different concentrations of LMP1-ΔT were co-incubated with two mutants of ANT1 at room temperature for 30 min and then subjected to 19F-NMR analysis. As the concentration of LMP1-ΔT increased, the fluorine state did not change, confirming that the binding of LMP1 to ANT1 is necessary for the conformational change of

ANT1(line 241-249). The results are shown in the figure below：

Figures 3H:

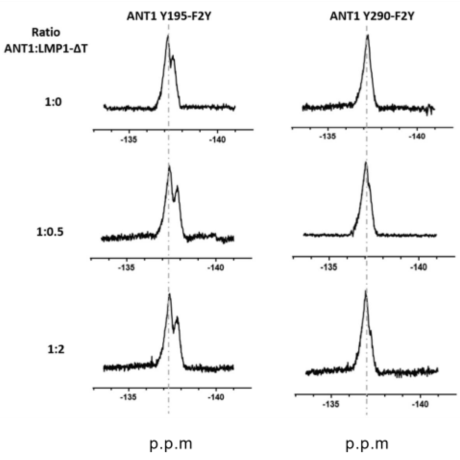

3. In Figure 3A, the results indicate that BKA, an inhibitor keeping ANT1 in m-state, inhibits the binding of ANT1 and VDAC1, while CATR, an inhibitor stabilizing ANT1 in c-state, promotes the binding. But the authors drew a totally opposite conclusion in the manuscript (line 211-214), which should be corrected.

Author Response: Revised in the revised manuscript: To test this hypothesis, HK1 cells were treated with different concentrations of BKA or CATR for 24 hours, and immunoprecipitation results demonstrated that BKA inhibits the binding of ANT1 to VDAC1, whereas CATR promotes the binding (line 216-218).

4. In Figure 3F, this reviewer would expect that the cell viability in BKA treatment groups is comparable with that in LMP1-overexprssed cells without BKA treatment because ANT1 is at m-state in these three groups, but it was not the case as shown in the results. Besides, it's better to use CATR to attenuate ANT1 at c-state to prove LMP1 modulate mitochondrial membrane potential and cell viability by keeping ANT1 at m-state.

Author Response: In LMP1-positive cells, there was no significant change in cell viability levels between the BKA-treated and untreated groups (Supplementary Figure 2F: Figure 3 in the original manuscript). We next treated LMP1-positive tumor cells with CATR and found a significant decrease in cell viability compared to the CATR-untreated group, demonstrating that LMP1 regulates cell viability by keeping

ANT1 in the m-state (Supplementary Figure 2F) (line 250-255).

Supplementary Figure 2F:

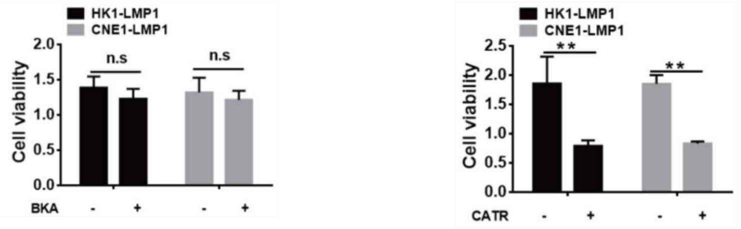

5. The authors should check through the manuscript carefully to correct typo errors and other mistakes. Following are some examples:

1) The figure legend of Fig 2E indicated that the experiment was carried out in 293T, but the results shown in the figure were not in 293T;

Author Response: The results in Figure 2D (Figure 2E in the original manuscript) were experimented in LMP1-positive cells and have been modified in the revised manuscript (line 183-185).

2) The antibodies used for IP in Fig 2F and Fig 2G are reversed, making the results confusing;

Author Response: The antibody labeling in Figure 2F and Figure 2G has been modified and the results are shown in the following figures:

Figures 2F:

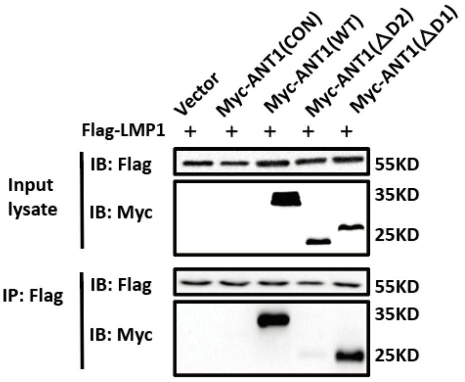

Figures 2G:

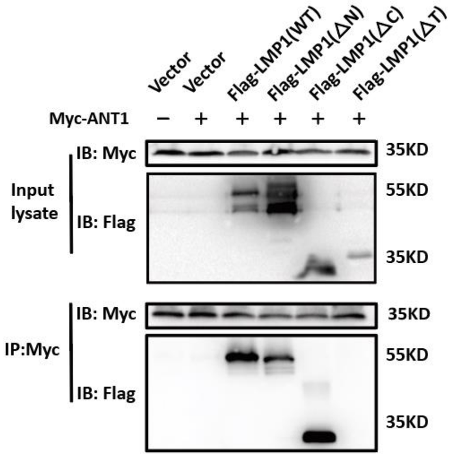

3) Label "G" was missing and label "F" was in a wrong place in Figure 2;

Author Response: Corrections have been made in the revised draft.

4) "7-ADD" should be corrected as "7-AAD" in Fig 5G and the manuscript;

Author Response: Corrections have been made in the revised draft (line 325-329).

5) The results and methods of Figure 6 showed that the xenograft experiment used up to 5×10^7 cells, which was far beyond common sense. Please check through the texts to correct these mistakes and make sure that the figure legends and methods contain enough information to allow interpretation and replication of the results.

Author Response: The number of HK1-LMP1 cells inoculated subcutaneously was 5×10^6 per mouse. Corrected in the revised manuscript (line 630-631).

Reviewer #3:

Lin Zhao and colleagues aimed to identify mechanism by which a latent membrane protein 1 (LMP1), encoded by Epstein-Barr virus (EBV), induces alteration of ANT1 conformation in cisplatin (CDDP)-resistant nasopharyngeal carcinoma cells (NPC). The authors described that EBV-LMP1 inhibits the mPTP opening by binding to ANT1, leading to the increased tumour cell survival and drug resistance. ANT1 inhibitor CATR and especially its combination with CDDP improved a chemosensitivity of EBV-LMP1-positive cells. In my opinion several obtained results not necessarily support the made conclusion, the interpretation of several experiments is not convincing and important controls are lacking. The manuscript itself reminds me of the first draft.

Major comments

1. The introduction describes the current research field very narrowly and subjectively. The fact that the ANT1 is a part of mPTP is highly controversial. Zhao et al. didn´t mention the existing hot debates and other hypotheses, excluding ANT1. ANT1 was shown to be not only ATP/ADP transporter, but obviously also participates in the

proton transport in the presence of fatty acids. As recently shown, the proton transport is also affected by CATR and BA.

Author Response: As suggested by the reviewers, we have fleshed out the research area of ANT in the Frontiers section as appropriate: ANT is not only an ATP/ADP transporter, but apparently is also involved in proton transport in the presence of fatty acids; and the molecular composition of mPTP is not fully understood at this time and remains an area of debate, with ANT and F0F1-ATP synthase being the main competitors for its composition (line 64-67/71-74).

2. Several very important conclusions are based on the measurements of mitochondrial

membrane potential (MMP) using fluorescent dyes TMRM and JC1. However, only measurements which investigate the immediate MMP changes after direct addition of substances potential are easy to interpret. The comparison of two different cell cultures (e.g. wt and KO or cells before and after 24 h incubation (Fig. 5 B/C)) is very challenging because of the different dye/cell concentration, change in mitochondrial surface/volume ratio and loading time.

Author Response: In the process of measuring mitochondrial membrane potential, to determine whether there is an effect of dye/cell concentration, loading time, etc. on the mitochondrial membrane potential. According to the reviewer's suggestion, we detected the mitochondrial membrane potential by adding BKA and CATR immediately after 24 h of cisplatin treatment of LMP1-positive NPC cells (Supplementary Figure 3B/C), and the mitochondrial membrane potential results showed the same trend as 24 h (Figure 5B, Supplementary Figure 3D) before adding BKA and CATR. The results are shown in the following figures:

Supplementary Figure 3B:

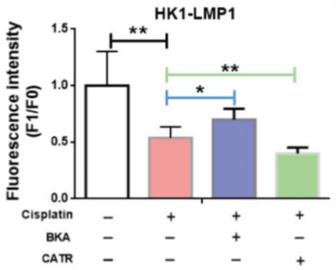

Supplementary Figure 3C:

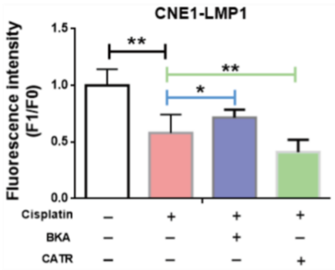

Figure 5B:

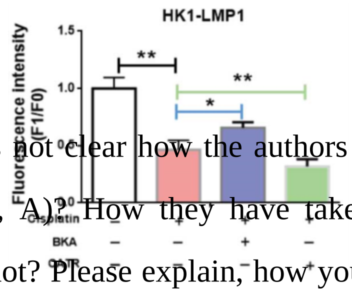

Supplementary Figure 3D:

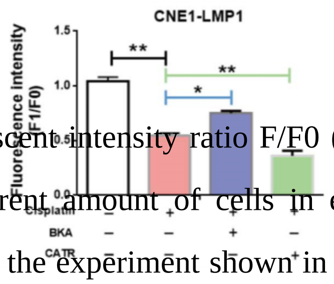

3. It is not clear how the authors calculated the fluorescent intensity ratio F/F0 (e.g. Fig. 1, A)? How they have taken in account a different amount of cells in each snapshot? Please explain, how you perform statistics in the experiment shown in Fig. 1, D? My concern is that the fluorescence will depend on the cell amount.

Author Response: The average fluorescence value of each field of view was obtained by ImageJ software to derive the fluorescence intensity ratio F/F0; average fluorescence value = overall fluorescence intensity per field of view/number of cells per field of view, and at least 6 different fields of view were taken for each experiment for statistical analysis.

4. The scientific quality of the figures and their descriptions is below the usual requirements for a bachelor thesis. Sometimes I get the impression that the authors do not know exactly what they are measuring. This applies to all Figures. I will give only some examples below.

Fig. 1, B: y-axis is entitled scientifically incorrect!

Author Response: Changes have been made in the rework and the results are shown in the figure below:

Figure 1B:

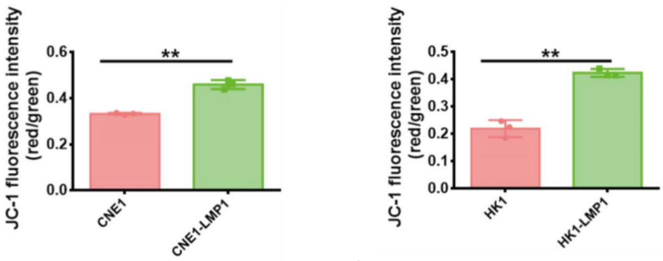

Fig. 1, C: x-axis is entitled scientifically incorrect!

Author Response: Changes have been made in the rework and the results are shown in the figure below:

Figure 1C:

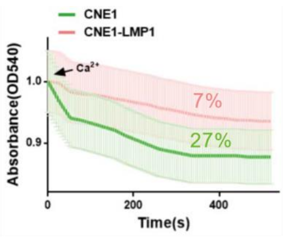

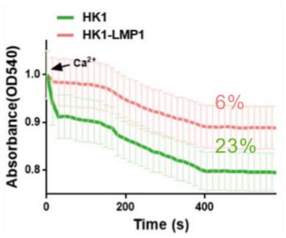

Line 141 I don´t see how the conclusion "suggesting that EBV-LMP1 ..... increases the mitochondrial membrane potential" can be made from Fig. 1C.

Author Response: Mitochondria underwent swelling mainly caused by mPTP opening. The swelling rate of HK1-LMP1 (6%) shown in Figure 1C was significantly lower than that of HK1 (23%), and similar conclusions were obtained in CNE1 (27%) /CNE1-LMP1 (7%). Combining the results in Figure 1A/B, we confirmed that LMP1 can increase the mitochondrial membrane potential.

Figure 1:

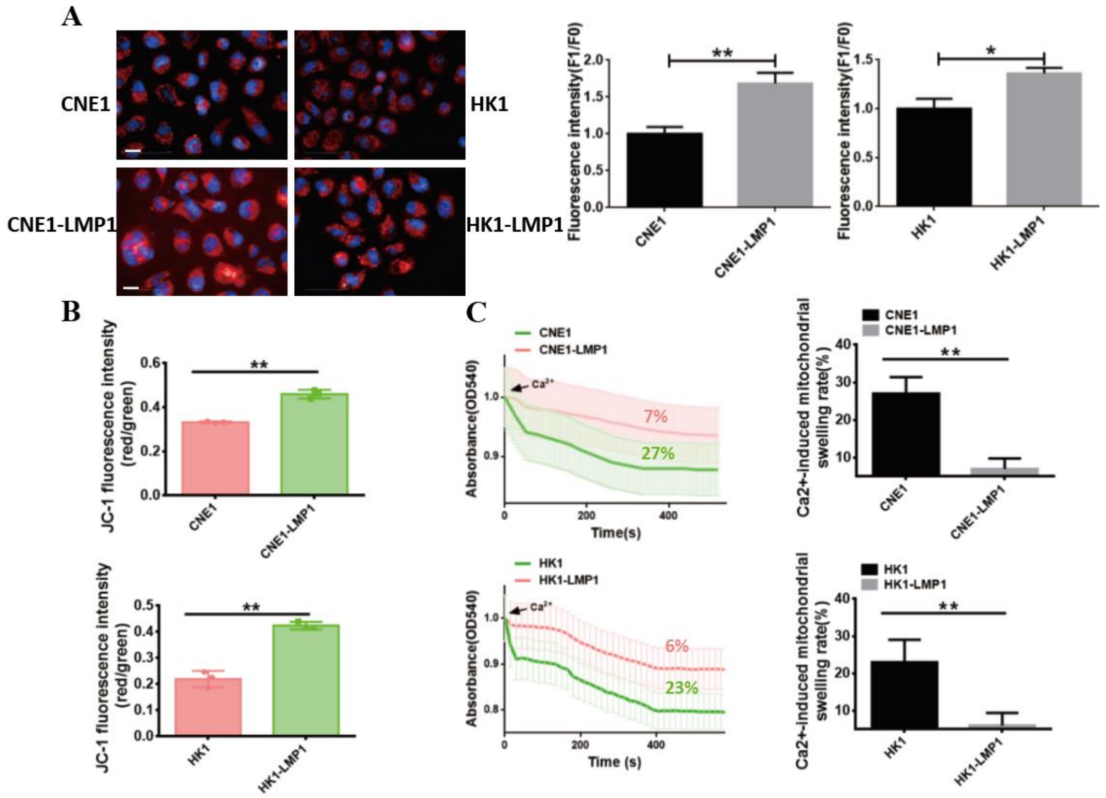

The experiment shown in Fig. 1D should be explain better. The use of ionomycin is controversial (PMID: 28655872), the authors should take better A23187, to induce a decrease in calcein fluorescence.

Author Response: The revised manuscript replaced ionomycin with A23187 to induce a reduction in calreticulin fluorescence, and the results are shown in the

following figure:

Figure 1D:

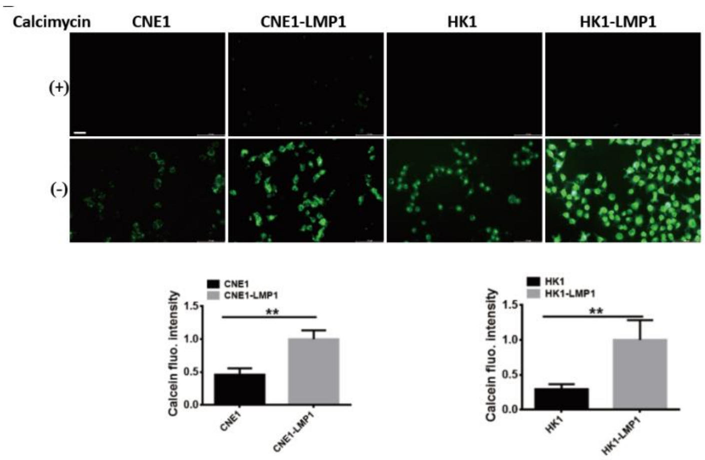

Suppl. Fig. 1 Actin is not appropriately shown in Western Blots and cannot be used as a control.

Author Response: The WB experiment in Supplementary Figure 1A used whole cell lysates, and VDAC1 is one of the components of mPTP, so it is more appropriate to use actin as an internal reference here.

Please explain what we see in the Western Blot (?) at 35 kD. There is no appropriate description of the Fig. 3D.

Author Response: Fig. 3F (line 1001) in 35KD is the position of western marker, the molecular weight of ANT1 is roughly around 32KD, and the molecular weight of GST-ANT1 is slightly larger than 32KD.

Suppl. Fig. 2C. The line description: "19F-NMR assay of ANT1-WT" doesn´t help to understand what was measured.

Author Response: The use of NMR detection of ANT1-WT in Suppl. Fig. 2C is used as a reference; ANT1-WT not doped with difluorotyrosine which is unable to detect the fluorine signal in NMR.

5. In several experiments the antibodies (AB) against ANT1 and ANT2 are used. Due to relatively high homology between mitochondrial transporters AB against ANT1 are known to be highly unspecific and cannot be used in immunohistochemical staining. Authors should perform (i) full size WB for ANT1 expression in HK1 cell to validate the use of the antibody for staining and (ii) to show the specificity of this AB using ANT1 KO cells/tissues as negative control. Also appropriate positive control (e.g. recombinant ANT1) is required.

Author Response: We confirmed the specificity of ANT1 antibody by both prokaryotic and eukaryotic methods: first, we expressed HK1 cell-derived ANT1 in prokaryotic cells, purified it and detected it by ANT1 antibody (Supplementary Figure 1.C); second, we overexpressed and knocked down ANT1 in HK1 cells and then detected ANT1 expression by WB (Supplementary Figure 1.D).

Supplementary Figure 1C:

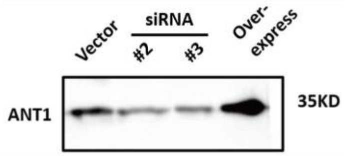

Supplementary Figure 1.D:

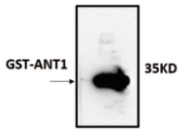

6. Which absolute values for ATP/ADP exchange were measured in the experiments shown in Fig. 4b? How this exchange was measured?

Author Response: Absolute values of the ATP-ADP exchange rate can be obtained (as shown in Supplementary Table 1); and the ATP-ADP exchange rate was measured as previously described (Kawamata, Starkov et al., 2010, Zhang et al., 2017).

Supplementary Table 1:

Table 1: ADP/ATP exchange rate in NPC cells (expressed as nmol/s).

Groups	Vehicle	BKA	CATR
CNE1	5.3±2.3	0.62±0.02	0.72±0.01
CNE1-LMP1	2.2±0.92	0.73±0.04	0.86±0.03
HK1	6.8±1.2	0.61±0.03	0.65±0.01
HK1-LMP1	3.7±0.98	0.74±0.04	0.60±0.02

Data were expressed as Mean±SD, n=5.

7. Please explain how the Seahorse experiment justifies the conclusion "the process is closely related to ANT1 conformation-based functional restrictions." (Fig. 4, C).

Author Response: ANT1 enables mitochondrial ADP/ATP exchange through uninterrupted switching of its c- and m-state conformations. BKA and CATR are conformational inhibitors of ANT1 (BKA puts ANT1 in m-stae; CATR puts ANT1 in c-stae). In Figure 4C/D the maximal respiration of BKA and CATR-treated HK1 cells was significantly lower than that with the control group, with similar findings in other HK1-LMP1/CNE1/CNE1-LMP1.

Figure 4C:

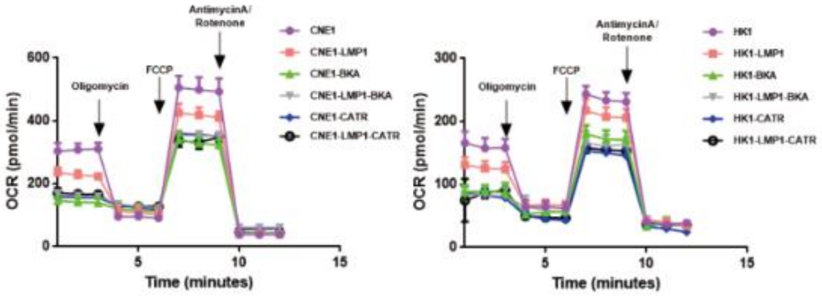

Figure 4D:

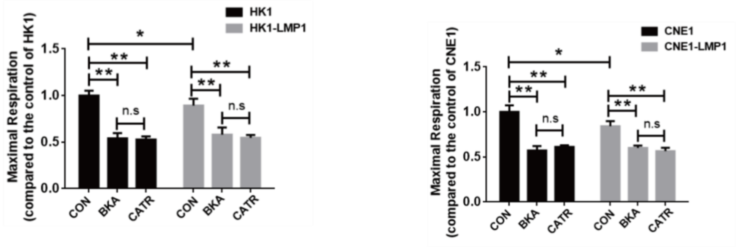

8. What is the hypothesis for the usage of two sets of NPC cells?

Author Response: LMP1 is an important latent membrane protein encoded by the oncogenic virus EBV that has been shown to be involved in the development of a variety of tumors, and more than 95% of patients with nasopharyngeal carcinoma are EBV positive (Cao Y, Signal Transduct Target Ther. 2021), so we selected two representative pairs of NPC cells, CNE1 and HK1, as our study subjects.

9. A representative IP for LPM1 (Fig. 3 B) is not convincing. Authors should show at least two other experiments.

Author Response: First, we confirmed by IP that LMP1 functions similarly to BKA

to maintain ANT1 in the m-state (Fig. 3 A/C), and next we directly confirmed by 19-NMR that LMP1 induces conformational changes in ANT1 and that the binding of LMP1 to ANT1 is necessary for its occurrence (Fig. 3 G/H).

Fig. 3A:

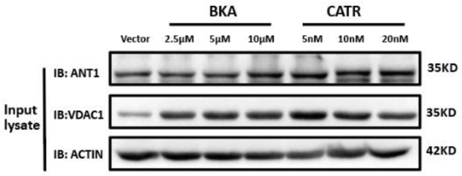

Fig. 3C:

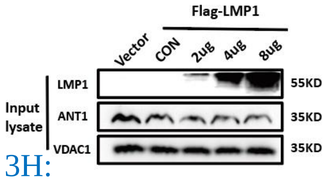

Fig. 3G:

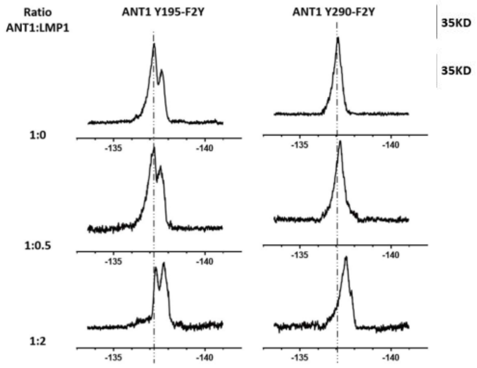

Fig. 3H:

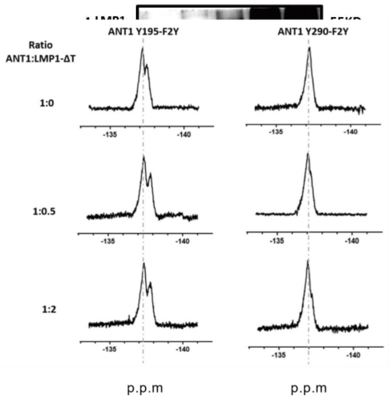

10. CDDP (cis-diamminedichloroplatinum? this abbreviation is not explained in the text) and cisplatin are obviously the same substances. However, authors describe their experiment like this (Line 291): "... cells were divided into a control group, CDDP-treatment only group, cisplatin-treatment only group, and a combination of CDDP and cisplatin-treated group, respectively." This description should be corrected.

Author Response: Revised were highlighted in the manuscript: In order to verify the biological effect of CATR and cisplatin in EBV-LMP1 NPC cells, cells were divided into a control group, CATR-treatment only group, CDDP-treatment (cisplatin treatment group) only group, and a combination of CDDP and CATR-treated group, respectively (line 317-319).

9th Sep 2021

Dear Prof. Cao,

Thank you for the submission of your revised manuscript to EMBO Molecular Medicine.
We have received the enclosed report from referees #1 and #2, and as you will see, they are now supportive of publication. I am therefore pleased to inform you that we will be able to accept your manuscript once the following minor editorial points will be addressed:

1/ Please address the minor comment from referee #1.

2/ Main manuscript text:
- Please address the queries from our data editors in track changes mode in the main manuscript file labelled 'Data edited MS file'. Please use this file for any further modification and only keep in track changes mode the new modifications.
- During our standard cross-check analysis, text similarities with previously published materials from your lab were found (see screenshots attached), therefore please slightly modify these paragraphs. Please also make sure that figure legends are sufficiently different from previously published materials. Please note that Material and methods is not considered for plagiarism check.
- Please remove the highlights in the text.
- Material and methods:
o Cells: please indicate whether the cells are of mouse or human origin, and whether they were authenticated and tested for mycoplasma contamination.
o Please provide information regarding antibodies (references and dilutions).
o Please describe the housing and husbandry conditions for the mice.
o In your manuscript file, you indicated:
"The materials and methods for coimmunoprecipitation, CCK-8, NMR, mitochondrial swelling, mitochondrial membrane potential, flow cytometry, ATP experiments, determination of the mitochondrial stress, TUNEL assays, PLAs, and plasmid transfection are provided in Supplementary Materials." However, I could not find this file. Please clarify.
- Data Availability section: Please provide the accession numbers for your PDB dataset in this section (and URL). Note that the Data Availability Section is restricted to new primary data that are part of this study.
- References: Please reformat to have 10 authors listed before et al.

3/ Figures and Appendix:
- Please indicate in legends exact n= and exact p= values, not a range, (including for non significant p values, ns). Some people found that to keep the figures clear, providing a supplemental table with all exact p-values was preferable. You are welcome to do this if you want to.
- Supplementary Figure 2F is not referenced in the text, please correct.
- Suppl. Table 1 should be renamed Table 1, and Suppl. Fig 1-3 should be renamed Figures EV1-3.

4/ Checklist:
- Please provide more information in part B/5
- Please provide information on antibodies and cells (reference, dilutions, cell source, mycoplasma test) in part C.
- Please fill parts D/8-9 and F/18-19.

5/ The paper explained: EMBO Molecular Medicine articles are accompanied by a summary of the articles to emphasize the major findings in the paper and their medical implications for the non-specialist reader. Please provide a draft summary of your article highlighting
- the medical issue you are addressing,
- the results obtained and
- their clinical impact.

This may be edited to ensure that readers understand the significance and context of the research. Please refer to any of our published articles for an example.

6/ Every published paper now includes a 'Synopsis' to further enhance discoverability. Synopses are displayed on the journal webpage and are freely accessible to all readers. They include a short stand first (maximum of 300 characters, including space) as well as 2-5 one-sentences bullet points that summarizes the paper. Please write the bullet points to summarize the key NEW findings. They should be designed to be complementary to the abstract - i.e. not repeat the same text. We encourage inclusion of key acronyms and quantitative information (maximum of 30 words / bullet point). Please use the passive voice. Please attach these in a separate file or send them by email, we will incorporate them accordingly.

Please also suggest a striking image or visual abstract to illustrate your article as a PNG file 550 px wide x 300-600 px high.

7/ As part of the EMBO Publications transparent editorial process initiative (see our Editorial at http://embomolmed.embopress.org/content/2/9/329), EMBO Molecular Medicine will publish online a Review Process File (RPF) to accompany accepted manuscripts.
This file will be published in conjunction with your paper and will include the anonymous referee reports, your point-by-point response and all pertinent correspondence relating to the manuscript. Let us know whether you agree with the publication of the RPF and as here, IF YOU WANT TO REMOVE OR NOT any figures from it prior to publication.
Please note that the Authors checklist will be published at the end of the RPF.

I look forward to receiving your revised manuscript.

Yours sincerely,

Lise Roth

Lise Roth, PhD
Editor
EMBO Molecular Medicine

To submit your manuscript, please follow this link:

https://embomolmed.msubmit.net/cgi-bin/main.plex

***** Reviewer's comments *****

Referee #1 (Comments on Novelty/Model System for Author):

The authors addressed most of my concerns, as well as those uttered by the other two reviewers.

Referee #1 (Remarks for Author):

The word "armpit" should be deleted at the proof stage.

Referee #2 (Remarks for Author):

The authors have fully addressed my concerns. I have no further questions.

The authors performed the requested editorial changes.

29th Sep 2021

Dear Prof. Cao,

We are pleased to inform you that your manuscript is accepted for publication and is now being sent to our publisher to be included in the next available issue of EMBO Molecular Medicine.

Please note that I have replaced the TPE in the manuscript as discussed previously:

Problem
The Epstein-Barr virus (EBV) encodes the key oncogenic protein LMP1, which plays an important role in promoting resistance to therapy. In this study, we explored novel targets and potential mechanisms by which EBV-LMP1 regulates resistance to cisplatin in nasopharyngeal carcinoma (NPC).
Results
We found that EBV-LMP1 can localize to the mitochondria to bind directly to adenine nucleotide translocase-1 (ANT1), fixing the ANT1 conformation in the m-state, thereby increasing the mitochondrial membrane potential and promoting the viability of NPC cells. Carboxyatractyloside (CATR), a conformational inhibitor of ANT1, which contributes to mPTP opening and cell death, enhanced the sensitivity of tumor cells to cisplatin.
Impact
Our study links for the first time ANT1 conformational changes to cisplatin chemosensitivity, highlighting the importance of protein conformational changes in tumor chemotherapy.

Should you not agree with this version, please let us know immediately. Please also carefully check the proofs once you receive them, as mistakes might have happened between the many successive versions of the manuscript.

Congratulations on your interesting work!

With kind regards,

Lise Roth

Lise Roth, Ph.D
Scientific Editor
EMBO Molecular Medicine

Follow us on Twitter @EmboMolMed
Sign up for eTOCs at embopress.org/alertsfeeds

EMBO PRESS

YOU MUST COMPLETE ALL CELLS WITH A PINK BACKGROUND ➔

PLEASE NOTE THAT THIS CHECKLIST WILL BE PUBLISHED ALONGSIDE YOUR PAPER

Corresponding Author Name: Ya Cao	
Journal Submitted to: EMBO Molecular Medicine	
Manuscript Number: EMM -2021-14072	

Reporting Checklist For Life Sciences Articles (Rev. June 2017)

This checklist is used to ensure good reporting standards and to improve the reproducibility of published results. These guidelines are consistent with the Principles and Guidelines for Reporting Preclinical Research issued by the NIH in 2014. Please follow the journal's authorship guidelines in preparing your manuscript.

A- Figures
1. Data
The data shown in figures should satisfy the following conditions:

- the data were obtained and processed according to the field's best practice and are presented to reflect the results of the experiments in an accurate and unbiased manner.
- figure panels include only data points, measurements or observations that can be compared to each other in a scientifically meaningful way.
- graphs include clearly labeled error bars for independent experiments and sample sizes. Unless justified, error bars should not be shown for technical replicates.
- if n< 5, the individual data points from each experiment should be plotted and any statistical test employed should be justified
- Source Data should be included to report the data underlying graphs. Please follow the guidelines set out in the author ship guidelines on Data Presentation.

2. Captions

Each figure caption should contain the following information, for each panel where they are relevant:

- a specification of the experimental system investigated (eg cell line, species name).
- the assay(s) and method(s) used to carry out the reported observations and measurements
- an explicit mention of the biological and chemical entity(ies) that are being measured.
- an explicit mention of the biological and chemical entity(ies) that are altered/varied/perturbed in a controlled manner.

- the exact sample size (n) for each experimental group/condition, given as a number, not a range;
- a description of the sample collection allowing the reader to understand whether the samples represent technical or biological replicates (including how many animals, litters, cultures, etc.).
- a statement of how many times the experiment shown was independently replicated in the laboratory.
- definitions of statistical methods and measures:
  - common tests, such as t-test (please specify whether paired vs. unpaired), simple χ2 tests, Wilcoxon and Mann-Whitney tests, can be unambiguously identified by name only, but more complex techniques should be described in the methods section;
  - are tests one-sided or two-sided?
  - are there adjustments for multiple comparisons?
  - exact statistical test results, e.g., P values = x but not P values < x;
  - definition of 'center values' as median or average;
  - definition of error bars as s.d. or s.e.m.

Any descriptions too long for the figure legend should be included in the methods section and/or with the source data.

In the pink boxes below, please ensure that the answers to the following questions are reported in the manuscript itself. Every question should be answered. If the question is not relevant to your research, please write NA (non applicable). We encourage you to include a specific subsection in the methods section for statistics, reagents, animal models and human subjects.

USEFUL LINKS FOR COMPLETING THIS FORM

http://www.antibodypedia.com
http://1degreebio.org
http://www.equator-network.org/reporting-guidelines/improving-bioscience-research-repor

http://grants.nih.gov/grants/olaw/olaw.htm
http://www.mrc.ac.uk/Ourresearch/Ethicsresearchguidance/Useofanimals/index.htm
http://ClinicalTrials.gov
http://www.consort-statement.org
http://www.consort-statement.org/checklists/view/32-consort/66-title

http://www.equator-network.org/reporting-guidelines/reporting-recommendations-for-tumo

http://datadryad.org

http://figshare.com

http://www.ncbi.nlm.nih.gov/gap

http://www.ebi.ac.uk/ega

http://biomodels.net/

http://biomodels.net/miriam/
http://jjj.biochem.sun.ac.za
http://oba.od.nih.gov/biosecurity/biosecurity_documents.html
http://www.selectagents.gov/

B- Statistics and general methods

Please fill out these boxes ➔ (Do not worry if you cannot see all your text once you press return)

1.a. How was the sample size chosen to ensure adequate power to detect a pre-specified effect size?	It is determined according to different experimental purposes and reference to different literature
1.b. For animal studies, include a statement about sample size estimate even if no statistical methods were used.	In this study, the sample size for animal experiments was estimated in relation to the type of study design and the purpose of the experiment.
2. Describe inclusion/exclusion criteria if samples or animals were excluded from the analysis. Were the criteria pre-established?	NA
3. Were any steps taken to minimize the effects of subjective bias when allocating animals/samples to treatment (e.g. randomization procedure)? If yes, please describe.	We numbered the mice sequentially and grouped them using a randomized method.
For animal studies, include a statement about randomization even if no randomization was used.	Animal experiments were performed using randomized groups.
4.a. Were any steps taken to minimize the effects of subjective bias during group allocation or/and when assessing results (e.g. blinding of the investigator)? If yes please describe.	A double-blind method was used in this study.
4.b. For animal studies, include a statement about blinding even if no blinding was done	A double-blind method was used in this study.
5. For every figure, are statistical tests justified as appropriate?	Appropriate statistical tests are used according to the experimental data and purpose.The experimental data are presented as the mean values ± S.E.M. The statistical significance of the data was analyzed by using a standard student's t-test. A p-value of < 0.05 was deemed statistically significant and p< 0.01 was considered statistically significant.
Do the data meet the assumptions of the tests (e.g., normal distribution)? Describe any methods used to assess it.	NA
Is there an estimate of variation within each group of data?	NA

Is the variance similar between the groups that are being statistically compared?	Yes

C- Reagents

6. To show that antibodies were profiled for use in the system under study (assay and species), provide a citation, catalog number and/or clone number, supplementary information or reference to an antibody validation profile. e.g., Antibodypedia (see link list at top right), 1DegreeBio (see link list at top right).	Antibodies used included ANT1 mAb (ab110322, Abcam, UK, 1:1000), VDAC1 mAb (Ab34726, Abcam, UK, 1:1000), HSP60 mAb (Sc-1052, Santa Cruz Biotechnology, USA, 1:1000), β-actin mAb (A5441, Sigma, Germany, 1:10000), cyto C mAb (556433, BD, USA, 1:1000), TIM23 mAb (11123-1-AP, Proteintech, USA, 1:1000), ANT2 mAb (14671S, CST, USA, 1:2000), VDAC2 mAb (11663-1-AP, Proteintech, USA, 1:1000), caspase-9 mAb (9502S, CST, USA, 1:1000), Flag mAb (F1804, Sigma-Aldrich, USA, 1:1000), and cleaved caspase-3 mAb (9664S, CST, USA, 1:1000).
7. Identify the source of cell lines and report if they were recently authenticated (e.g., by STR profiling) and tested for mycoplasma contamination.	CNE1 purchased from the Cell Resource Center of Central South University; HK1 gift from Prof Sai-Wah Tsao, The University of Hong Kong. CNE1-LMP1 and HK1-LMP1 are stably LMP1 expressing human nasopharyngeal carcinoma cells. All cells were validated by STR analysis and tested regularly for mycoplasma.

* for all hyperlinks, please see the table at the top right of the document

D- Animal Models

8. Report species, strain, gender, age of animals and genetic modification status where applicable. Please detail housing and husbandry conditions and the source of animals.	Female BALB/c-nude mice (5–6 weeks) were purchased from SLAC Laboratory Animal Co. Ltd. (Changsha, China). The Department of Zoology of Central South University has a well-equipped animal house of SPF level, using facilities of 3300 square meters, including nearly 8000 cage spaces for mice and more than 1200 cage spaces for rats, equipped with ultra-clean bench and anesthesia machine. The purchase and feeding of experimental animals are in accordance with national standards, ensuring the smooth development of animal experiments.
9. For experiments involving live vertebrates, include a statement of compliance with ethical regulations and identify the committee(s) approving the experiments.	The in vivo study was approved by the Medical Ethics Committee (for experimental animals) of the Xiangya Hospital, Central South University.
10. We recommend consulting the ARRIVE guidelines (see link list at top right) (PLoS Biol. 8(6), e1000412, 2010) to ensure that other relevant aspects of animal studies are adequately reported. See author guidelines, under 'Reporting Guidelines'. See also: NIH (see link list at top right) and MRC (see link list at top right) recommendations. Please confirm compliance.	Confirmation of compliance.

E- Human Subjects

11. Identify the committee(s) approving the study protocol.	NA
12. Include a statement confirming that informed consent was obtained from all subjects and that the experiments conformed to the principles set out in the WMA Declaration of Helsinki and the Department of Health and Human Services Belmont Report.	NA
13. For publication of patient photos, include a statement confirming that consent to publish was obtained.	NA
14. Report any restrictions on the availability (and/or on the use) of human data or samples.	NA
15. Report the clinical trial registration number (at ClinicalTrials.gov or equivalent), where applicable.	NA
16. For phase II and III randomized controlled trials, please refer to the CONSORT flow diagram (see link list at top right) and submit the CONSORT checklist (see link list at top right) with your submission. See author guidelines, under 'Reporting Guidelines'. Please confirm you have submitted this list.	NA
17. For tumor marker prognostic studies, we recommend that you follow the REMARK reporting guidelines (see link list at top right). See author guidelines, under 'Reporting Guidelines'. Please confirm you have followed these guidelines.	NA

F- Data Accessibility

18: Provide a "Data Availability" section at the end of the Materials & Methods, listing the accession codes for data generated in this study and deposited in a public database (e.g. RNA-Seq data: Gene Expression Omnibus GSE39462, Proteomics data: PRIDE PXD000208 etc.) Please refer to our author guidelines for 'Data Deposition'.<br><br>Data deposition in a public repository is mandatory for:<br>a. Protein, DNA and RNA sequences<br>b. Macromolecular structures<br>c. Crystallographic data for small molecules<br>d. Functional genomics data<br>e. Proteomics and molecular interactions	The datasets produced in this study are available in the following databases:<br>● https://www.rcsb.org/structure/4C9H<br>● https://www.rcsb.org/structure/6GCI
19. Deposition is strongly recommended for any datasets that are central and integral to the study; please consider the journal's data policy. If no structured public repository exists for a given data type, we encourage the provision of datasets in the manuscript as a Supplementary Document (see author guidelines under 'Expanded View' or in unstructured repositories such as Dryad (see link list at top right) or Figshare (see link list at top right).	Important datasets for this study are provided in the form of source datas in the manuscript.
20. Access to human clinical and genomic datasets should be provided with as few restrictions as possible while respecting ethical obligations to the patients and relevant medical and legal issues. If practically possible and compatible with the individual consent agreement used in the study, such data should be deposited in one of the major public access-controlled repositories such as dbGAP (see link list at top right) or EGA (see link list at top right).	NA
21. Computational models that are central and integral to a study should be shared without restrictions and provided in a machine-readable form. The relevant accession numbers or links should be provided. When possible, standardized format (SBML, CellML) should be used instead of scripts (e.g. MATLAB). Authors are strongly encouraged to follow the MIRIAM guidelines (see link list at top right) and deposit their model in a public database such as Biomodels (see link list at top right) or JWS Online (see link list at top right). If computer source code is provided with the paper, it should be deposited in a public repository or included in supplementary information.	NA

G- Dual use research of concern

22. Could your study fall under dual use research restrictions? Please check biosecurity documents (see link list at top right) and list of select agents and toxins (APHIS/CDC) (see link list at top right). According to our biosecurity guidelines, provide a statement only if it could.	NO
